# Characterization of super‐enhancer‐associated functional lncRNAs acting as ceRNAs in ESCC

**DOI:** 10.1002/1878-0261.12726

**Published:** 2020-06-20

**Authors:** Qiu‐Yu Wang, Liu Peng, Yang Chen, Lian‐Di Liao, Jia‐Xin Chen, Meng Li, Yan‐Yu Li, Feng‐Cui Qian, Yue‐Xin Zhang, Fan Wang, Chun‐Quan Li, De‐Chen Lin, Li‐Yan Xu, En‐Min Li

**Affiliations:** ^1^ The Key Laboratory of Molecular Biology for High Cancer Incidence Coastal Chaoshan Area Shantou University Medical College Shantou China; ^2^ School of Medical Informatics Harbin Medical University Daqing China; ^3^ Institute of Oncologic Pathology Medical College of Shantou University Shantou China; ^4^ Department of Medicine Cedars‐Sinai Medical Center Los Angeles CA USA

**Keywords:** cancer hallmark, CeRNA, long noncoding RNA, super‐enhancer

## Abstract

Long noncoding RNAs (lncRNAs) have important regulatory roles in cancer biology. Although some lncRNAs have well‐characterized functions, the vast majority of this class of molecules remains functionally uncharacterized. To systematically pinpoint functional lncRNAs, a computational approach was proposed for identification of lncRNA‐mediated competing endogenous RNAs (ceRNAs) through combining global and local regulatory direction consistency of expression. Using esophageal squamous cell carcinoma (ESCC) as model, we further identified many known and novel functional lncRNAs acting as ceRNAs (ce‐lncRNAs). We found that most of them significantly regulated the expression of cancer‐related hallmark genes. These ce‐lncRNAs were significantly regulated by enhancers, especially super‐enhancers (SEs). Landscape analyses for lncRNAs further identified SE‐associated functional ce‐lncRNAs in ESCC, such as HOTAIR, XIST, SNHG5, and LINC00094. THZ1, a specific CDK7 inhibitor, can result in global transcriptional downregulation of SE‐associated ce‐lncRNAs. We further demonstrate that a SE‐associated ce‐lncRNA, LINC00094 can be activated by transcription factors TCF3 and KLF5 through binding to SE regions and promoted ESCC cancer cell growth. THZ1 downregulated expression of LINC00094 through inhibiting TCF3 and KLF5. Our data demonstrated the important roles of SE‐associated ce‐lncRNAs in ESCC oncogenesis and might serve as targets for ESCC diagnosis and therapy.

## Graphical abstract

We developed the GloceRNA method for the identification of functional ce‐lncRNAs based on merging global and local regulatory direction consistency of expression associated with ceRNAs. GloceRNA identified many known and novel functional ce‐lncRNAs, which regulated the expression of a large number of cancer hallmark genes. We further identified novel SE‐associated ce‐lncRNAs and demonstrated their important roles in ESCC.

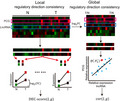

**Abbreviations**
Ce‐lncRNAs: LncRNAs acting as ceRNAs
CeRNAs: competing endogenous RNAs
ChIP‐seq: chromatin immunoprecipitation sequencing
ESCC: esophageal squamous cell carcinoma
GloceRNA: global and local regulatory direction consistency of expression of ceRNAs
LncRNAs: long noncoding RNAs
OS: overall survival
PCGs: protein‐coding genes
qRT‐PCR: quantitative real‐time PCR
SEs: super‐enhancers
siRNA: small interfering RNA
TEs: typical enhancers

## 1 Introduction

Long noncoding RNAs (lncRNAs) participate in a wide range of biological and cellular processes through mechanisms including modulation of chromatin structure, scaffolding, mRNA stability, or other transcriptional and post‐transcriptional processes (Flynn and Chang, 2014; Gupta *et al*., 2010; Schmitt and Chang, 2016; Vance and Ponting, 2014). Although some lncRNAs have well‐characterized biological functions, the vast majority of this class of molecules remains functionally uncharacterized (Batista and Chang, 2013; Du *et al*., 2013; Hosono *et al*., 2017; Li *et al*., 2018; Prensner *et al*., 2011, 2013; Zhang *et al*., 2018a, 2018d,2018a, 2018d). Accumulating evidence predicts that a large number of lncRNAs may act as competing endogenous RNAs (ceRNAs) to sponge miRNAs, resulting in the derepression of miRNA targets (Conte *et al*., 2017; Karreth and Pandolfi, 2013; Paci *et al*., 2014; Salmena *et al*., 2011; Tay *et al*., 2014; Zhou *et al*., 2016). The ceRNA mechanisms might be general acting in downstream regulation of lncRNAs (Paci *et al*., 2014; Poliseno *et al*., 2010). Thus, it is of great interest to uncover functional lncRNAs through characterizing lncRNAs acting as ceRNAs (ce‐lncRNAs). Indeed, studies demonstrated that previously uncharacterized lncRNAs could be functionalized, partly through the identification of their ceRNA interactors, and presented a framework for the prediction and validation of ceRNA interactions (Cesana *et al*., 2011; Conte *et al*., 2017). Especially, Paci et al. proposed a novel and useful computational approach to identify lncRNAs to act as ceRNAs through calculating the difference between Pearson and partial correlation coefficients (Paci *et al*., 2014). Based on the approach, they effectively explored miRNA decoy mechanism in gene regulatory circuitry using expression data from breast invasive carcinoma.

Enhancers are cis‐acting DNA segments that control cell type‐specific gene expression. Locally clustered enhancers form super‐enhancers (SEs), which are enriched for binding of a large number of transcription factors and play prominent roles in control of gene expression program and cell identity (Amaral and Bannister, 2014; Chipumuro *et al*., 2014; Hnisz *et al*., 2013; Whyte *et al*., 2013). Importantly, SEs exhibit much stronger lineage and tissue specificity compared with typical enhancers (TEs) (Hnisz *et al*., 2013). Because SEs are frequently identified near protein‐coding genes (PCGs) or noncoding RNAs that are important for controlling cell identity and differentiation, characterizing the function of SEs provides an opportunity to quickly identify key nodes driving diseases and biological processes (Hnisz *et al*., 2013, 2015; Jiang *et al*., 2019; Qian *et al*., 2019; Tang *et al*., 2019). Recently, some enhancer databases were developed, including SEdb (Jiang *et al*., 2019), db‐SUPER (Khan and Zhang, 2016), SEA (Wei *et al*., 2016), and ENdb (Bai *et al*., 2020). These databases provided a large number of SE/TE regions and related annotation information for various tissue/cell types. SE‐associated upstream and downstream regulatory analysis can be further performed using the SEanalysis and KnockTF tool, which characterized SE‐associated genes and transcription factors binding to target SEs (Feng *et al*., 2020; Qian *et al*., 2019). Studies have shown that a large number of novel noncoding RNAs are capable of being driven by SEs/TEs (Duan *et al*., 2016; Hnisz *et al*., 2013; Huang *et al*., 2019; Jiang *et al*., 2018b; Miao *et al*., 2018; Peng *et al*., 2019; Wood *et al*., 2018; Xiang *et al*., 2014; Xie *et al*., 2018; Zhang *et al*., 2017). Especially, a few SE‐associated lncRNAs have well‐characterized functions in cancer (Jiang *et al*., 2018b; Peng *et al*., 2019; Xie *et al*., 2018), which reveals upstream regulatory mechanisms of lncRNAs. For example, SE‐associated LncRNA LINC01503 was recently reported to promote the oncogenic phenotype of esophageal squamous cell carcinoma (ESCC) cells and was further identified as a squamous cell carcinoma‐specific lncRNA (Xie *et al*., 2018). SE‐associated LncRNA HCCL5 activated by transcription factor ZEB1 can promote the malignancy of hepatocellular carcinoma (Peng *et al*., 2019). Co‐activation of SE‐driven lncRNA CCAT1 by TP63 and SOX2 promotes squamous cancer progression (Jiang *et al*., 2018b). However, whether and how functional lncRNAs are regulated by SE‐associated genes is incompletely understood, due to the technical challenges in systematics characterization of SEs and functional lncRNAs. Since ce‐lncRNAs have high expression level, they might be controlled SEs/TEs, which perform important functions through regulating ce‐lncRNAs to driver a large of downstream target genes. Ce‐lncRNAs might appear to be a potential oncogenic downstream effector of SEs.

**Fig. 1:**
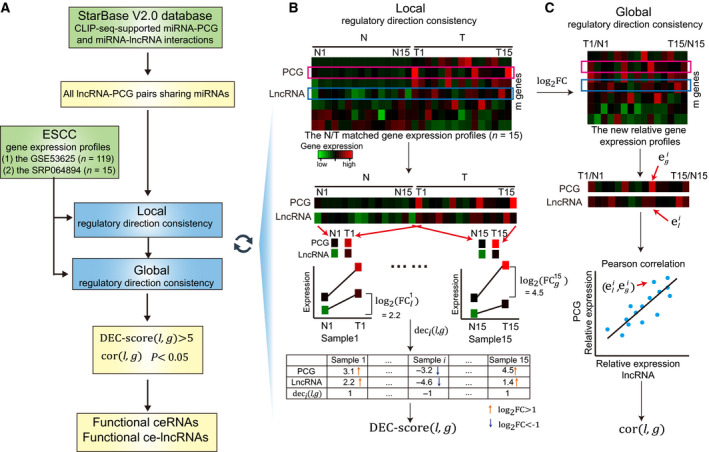
Schematic overview of the GloceRNA method. (A) Flow diagram of GloceRNA. The lncRNA‐PCG pairs sharing miRNA target sites are first established using CLIP‐seq‐supported miRNA‐PCG and miRNA‐lncRNA interactions. Next, GloceRNA calculates the local and global regulatory direction consistency of each lncRNA‐PCG pair. Finally, GloceRNA tests whether each lncRNA‐PCG pair meets the local and global direction consistency criteria. A lncRNA‐PCG pair sharing miRNAs will be identified as a functional ceRNA if it meets the two direction consistency criteria. The related lncRNA will be identified as a functional ce‐lncRNA. (B) Schematic overview of local regulatory direction consistency of expression of ceRNAs. (C) Schematic overview of global regulatory direction consistency of expression of ceRNAs. N, normal; T, tumor. DEC score(*l*, *g*): local regulatory direction consistency score of the lncRNA‐PCG pair, which can effectively evaluate possibility of ceRNAs significantly appearing in samples. dec*_i_*(*l*, *g*): the expression consistency score of the lncRNA‐PCG pair in the sample *i*, which represents the regulatory direction consistency of expression at single sample level. cor(*l*, *g*): global regulatory direction consistency score of the lncRNA‐PCG pair, which is calculated using Pearson correlation coefficient of lncRNA‐PCG. *P*: *P* value of Pearson correlation coefficient. $\log_{2}\left({\text{FC}}_{l}^{i}\right)$ and $\log_{2}\left({\text{FC}}_{g}^{i}\right)$: log_2_FC value of gene expression of lncRNA *l* and PCG *g* in sample *i*, which represent the relative gene expression level of tumor minus normal. $e_{l}^{i}=\log_{2}\left(FC_{l}^{i}\right)$ and $e_{g}^{i}=\log_{2}\left(FC_{g}^{i}\right)$.

Here, we developed a two‐stage computational approach, termed GloceRNA, for the identification of functional ce‐lncRNAs through combining global and local regulatory direction consistency of expression of ceRNAs (Fig. 1). We used normal/tumor (N/T) matched samples to improve prediction of functional ceRNAs. Especially, GloceRNA can measure the differential expression consistency of the lncRNA‐PCG pair at single sample level, which can effectively evaluate possibility of ceRNAs significantly appearing in some local samples. Using ESCC as a model, GloceRNA identified many known and novel functional ce‐lncRNAs. We demonstrated that GloceRNA robustly predicted ce‐lncRNAs in multiple ESCC datasets, and the predicted ce‐lncRNAs strongly regulated the expression of a large number of cancer hallmark genes. Moreover, we experimentally validated that some new predicted ce‐lncRNAs were highly associated with ESCC, including LINC00094, LINC00338, SNHG10, and MFI2‐AS1. Furthermore, we found that ce‐lncRNAs were significantly regulated by enhancers, especially SEs. We further demonstrated that a novel SE‐driven ce‐lncRNA – LINC00094 – promoted the growth and survival of ESCC cells. Lastly, we showed that TCF3 and KLF5 cooperatively regulated the express of LINC00094 through activation of its SE and promoter. Our study improved the original ceRNA identification methods, by using local regulatory direction consistency of expression strategy in N/T matched samples and emphasizing identification and analysis of functional ce‐lncRNAs in ESCC.

## 2 Materials and methods

### 2.1 Genome‐wide gene expression profiles of ESCC

Four datasets for genome‐wide gene expression profiles of ESCC were used in the study, including: (a) the GSE53625 (*n* = 119) dataset; (b) the SRP064894 dataset (*n* = 15); (c) the TCGA ESCC dataset (*n* = 80); (d) the GSE53625 (*n* = 60) dataset. The clinical and pathological characteristics of patients in all datasets were provided in Table S1 and Appendix S1. The GSE53625 dataset included two independent experimental subdatasets for gene expression profiles: GSE53625 (*n* = 119) and GSE53625 (*n* = 60). The GSE53625 (*n* = 119) dataset contained the 119 N/T matched samples. The GSE53625 (*n* = 60) dataset contained the 60 N/T matched samples. These data were downloaded from Gene Expression Omnibus (GEO) database (https://www.ncbi.nlm.nih.gov/geo/query/acc.cgi?acc=GSE53625). The expression profiles were performed using the agilent human lncRNA+mRNA Array v2.0 (4*180k) (Li *et al*., 2014a). To obtain maps from probes to annotated lncRNAs, we employed the blast program to map probes uniquely to the annotated lncRNA sequences. GENCODE (V19) and Ensembl 75 database were used as the reference annotation, and 8900 lncRNAs with at least unique probes mapped to it was used as its expression value. The SRP064894 dataset, which was generated by us, included the 15 N/T matched samples (Li *et al*., 2017). RNA sequencing was performed using the Illumina HiSeq 2500 (Illumina, San Diego, CA, USA). Sequencing reads were mapped to the human genome assembly (NCBI Build 37) using tophat (v2.0.6). The expression profiles with the lncRNAs and PCGs were extracted by using easyrnaseq (1.6.0). The TCGA ESCC dataset included the ESCC samples of 80 patients (Cancer Genome Atlas Research *et al*., 2017).

The GSE53625 (*n* = 119) and SRP064894 datasets were used as identifying functional lncRNA‐mediated ceRNAs and evaluating the robust of results. Further, the TCGA ESCC dataset was used as independent data to test the expression correlation of functional lncRNA‐mediated ceRNA pairs predicted by GloceRNA. Using the dataset, we also compared the expression correlation between functional lncRNA‐mediated ceRNA pairs and other potential ceRNA pairs sharing miRNAs. The GSE53625 (*n* = 119) and GSE53625 (*n* = 60) collected the survival information of patients. Therefore, they were used as survival analysis of functional ce‐lncRNAs and ceRNA pairs. Although the TCGA ESCC dataset also included the survival time of patients. However, the survival analysis were not performed in the study because survival time was too short for most of patients (the average survival time (day) = 193; 63% patients with survival time < 50 days, see Appendix S1).

### 2.2 CLIP‐seq‐supported miRNA‐mRNA interactions

Cross‐linking and Argonaute (Ago) immunoprecipitation coupled with high‐throughput sequencing (CLIP‐seq) could identify the genome‐wide interaction of miRNAs and their targets (37). The starBase V2.0 database is designed for decoding interaction network via integrating large‐scale CLIP‐seq (HITS‐CLIP, PAR‐CLIP, iCLIP, CLASH) data (Li *et al*., 2014b). MiRNA targets of starBase V2.0 were predicted by five target predicted algorithms, including TargetScan, miRanda, Pictar, PITA, and RNA22. In this study, we downloaded CLIP‐seq‐supported miRNA‐lncRNA and miRNA‐PCG interactions from starBase V2.0 database. In total, we obtained 423 975 miRNA‐PCG interactions with 386 miRNAs and 13 802 PCGs and 10 212 miRNA‐lncRNA interactions with 277 miRNAs and 1127 lncRNAs. All lncRNAs and PCGs, which can be assigned to HGNC symbol names, were used to the following ceRNA identification. The lncRNA‐PCG pairs sharing at least one miRNA were computed through considering CLIP‐seq‐supported miRNA‐PCG and miRNA‐lncRNA interactions from starBase V2.0 database. These pairs were used as identification of functional lncRNA‐mediated ceRNAs.

### 2.3 Identification of functional lncRNA‐mediated ceRNAs

We developed a computational approach, called GloceRNA, which aims to identify functional lncRNA‐mediated ceRNAs through combining global and local regulatory direction consistency of expression about ceRNAs (Fig. 1A). Notably, we first computed all lncRNA‐PCG pairs sharing miRNAs from CLIP‐seq‐supported miRNA‐PCG and miRNA‐lncRNA interactions from starBase V2.0 database. These pairs were used as the following identification of functional ceRNAs. Next, we calculated the local and global regulatory direction consistency of each lncRNA‐PCG pair. Finally, GloceRNA tested whether each lncRNA‐PCG pair meets the local and global direction consistency criteria. A lncRNA‐PCG pair sharing miRNAs will be identified as a functional ceRNA if it meets the criteria. The related lncRNA will be identified as a functional ce‐lncRNA.

We used N/T matched samples to evaluate local regulatory direction consistency of a potential lncRNA‐PCG ceRNA pair (Fig. 1B). We found that gene expression profiles with N/T matched samples are available for ESCC and many other diseases. Based on ceRNA principle, the increase of lncRNA expression in the lncRNA‐PCG ceRNA pair tends to lead to increase of the PCG expression, which means that the expression direction of ceRNA pair tends to be consistent. In N/T matched samples or even a single N/T matched sample, the expression direction of ceRNA pair also tends to be consistent. That is, for a pair of N/T samples from the same patient, a ceRNA pair usually displays consistently upregulated (or downregulated) in expression direction. Therefore, we used N/T matched samples to improve prediction of functional ceRNAs through capturing the local regulatory direction consistency information of expression. Suppose we have an ESCC expression profile dataset with *n* N/T matched samples and *m* genes (lncRNAs and PCGs) (Fig. 1B, Top panel). For a lncRNA‐PCG pair with lncRNA *l* and PCG *g*, the local regulatory direction consistency, called DEC score(*l*, *g*), can be measured using the expression level of lncRNA *l* and PCG *g*. We first compute the log_2_FC value of gene expression for the lncRNA *l* and PCG *g* in a N/T matched sample *i* (Fig. 1B, Middle panel) as follows:

(1) $$\log_{2}\left({\text{FC}}_{l}^{i}\right)=\log_{2}\left(y_{l}^{i}\right)-\log_{2}\left(x_{l}^{i}\right),$$

(2) $$\log_{2}\left({\text{FC}}_{g}^{i}\right)=\log_{2}\left(y_{g}^{i}\right)-\log_{2}\left(x_{g}^{i}\right),$$

where $y_{l}^{i}$ is the tumor expression value of lncRNA *l* in the N/T matched sample *i*, and $x_{l}^{i}$ is the normal expression value of the lncRNA in the N/T matched sample *i*. Similarly, $y_{g}^{i}$ and $x_{g}^{i}$ are the tumor and normal expression values of PCG *g* in the N/T matched sample *i*. The $\log_{2}\left({\text{FC}}_{l}^{i}\right)$ and $\log_{2}\left({\text{FC}}_{g}^{i}\right)$ values represent the relative gene expression level of tumor minus normal. Next, we used the log_2_FC values of the lncRNA *l* and PCG *g* to compute the differential expression consistency score dec*_i_*(*l*, *g*) of the lncRNA‐PCG pair at single sample level (Fig. 1B, Bottom panel). When two log_2_FC values of lncRNA and PCG are larger than 1 (i.e., $\log_{2}\left({\text{FC}}_{l}^{i}\right)$ > 1 and $\log_{2}\left({\text{FC}}_{g}^{i}\right)$ > 1), the lncRNA‐PCG pair will be defined as consistently upregulated (+1) in differential expression direction. On the contrary, the pair is defined consistently downregulated (−1) if all two values < −1. Therefore, the regulatory direction consistency of expression at single sample level was calculated as follows:

(3) $${\text{dec}}_{i}\left(l,g\right)=\left\{\begin{array}{cc} 1, & \text{if}\;\log_{2}\left(FC_{l}^{i}\right)>1\;\text{and}\;\log_{2}\left(FC_{g}^{i}\right)>1\\ -1, & \text{if}\;\log_{2}\left(FC_{l}^{i}\right)<-1\;\text{and}\;\log_{2}\left(FC_{g}^{i}\right)<-1\\ 0, & \text{otherwise} \end{array}\right.,$$

where dec*_i_*(*l*, *g*) is the expression consistency score of the lncRNA‐PCG pair in the sample *i*. The 1 and −1 represent that the lncRNA‐PCG pair is consistently upregulated or downregulated. For example, when $\log_{2}\left({\text{FC}}_{l}^{i}\right)$ = 3.1 and $\log_{2}\left({\text{FC}}_{g}^{i}\right)$ = 2.2, the value of dec*_i_*(*l*, *g*) is 1, which means that the lncRNA‐PCG pair is consistently upregulated (see Fig. 1B bottom panel and Table S2 for more examples). Finally, for a lncRNA‐PCG pair, we computed local regulatory direction consistency, called DEC score(*l*, *g*), through counting sum of all consistently up/downregulated samples across all *n* samples as follows:

(4) $$\text{DEC}\;-\;\text{score}\left(l,g\right)=\sum_{i=1}^{n}|{\text{dec}}_{i}\left(l,g\right)|,$$

DEC score(*l*, *g*) represents sample number that meets the differential expression direction consistency at single sample level (see Table S2 for an example of calculating DEC score(*l*, *g*)). DEC score(*l*, *g*) can be used to effectively evaluate possibility of ceRNAs significantly appearing in some local samples. The higher value of DEC score(*l*, *g*) is, the more samples meet that when a lncRNA is upregulated (or downregulated) in single ESCC sample, the corresponding PCG is also upregulated (or downregulated) in the same sample. Compared with global measures, the DEC score focused on mining ceRNA signals in the local samples since some strong ceRNA relationships may only exist in some patients due to cancer heterogeneity. In order to stably capture the local feature rather than global information, the cutoff of DEC score needs to be set appropriately. If the cutoff is set too small (e.g., < 3), we think that the result of ‘local’ regulatory direction consistency may be not stable due to random probability of regulatory direction consistency. On the contrary, too large cutoff (e.g., greater than half of the total number of samples) may lead to too strict, which makes DEC score tend to capture global rather than local information. In order to stably capture the local feature in two datasets, we think that the candidate cutoffs can be set as > 3, 4, 5, 6, 7, or 8, which may be more appropriate. We tested these cutoffs in two ESCC datasets (Table S3). In order to stably capture the local feature, and keep balance between local feature, number and similarity of ceRNAs in two datasets, the cutoff was set as > 5 in the paper. When DEC score(*l*, *g*) > 5, the pair is considered as meeting local regulatory direction consistency of ceRNAs.

For each lncRNA‐PCG pair sharing miRNAs, global regulatory direction consistency was further computed based on the relative gene expression profiles (Fig. 1C, Top panel). Notably, we first converted the gene expression profiles (*m* × 2*n* matrix) into a new gene expression profiles with relative expression level (*m* × *n* matrix) (Fig. 1C, Top panel). The log_2_FC values were used to represent the relative gene expression level of tumor minus normal in the new gene expression profiles. For example, the expression value of the lncRNA *l* and PCG *g* in the sample *i* of the new gene expression dataset is $\log_{2}\left({\text{FC}}_{l}^{i}\right)$ and $\log_{2}\left({\text{FC}}_{g}^{i}\right)$. Next, we used the Pearson correlation coefficient to evaluate the global regulatory direction consistency, called cor(*l*, *g*), of the lncRNA‐PCG pair based on relative expression values (log_2_FC) across all samples of the dataset (Fig. 1C, Middle panel).

(5) $$\text{cor}\left(l,g\right)=\frac{\sum_{i=1}^{n}\left(e_{l}^{i}-{\bar{e}}_{l}\right)\left(e_{g}^{i}-{\bar{e}}_{g}\right)}{\sqrt{\sum_{i=1}^{n}{\left(e_{l}^{i}-{\bar{e}}_{l}\right)}^{2}}\sqrt{\sum_{i=1}^{n}{\left(e_{g}^{i}-{\bar{e}}_{g}\right)}^{2}}},$$

where $e_{l}^{i}=\log_{2}\left({\text{FC}}_{l}^{i}\right)$ and $e_{g}^{i}=\log_{2}\left({\text{FC}}_{g}^{i}\right)$. The $e_{l}^{i}$ and $e_{g}^{i}$ are the relative expression levels of lncRNA *l* and PCG *g* in sample *i*. The ${\bar{e}}_{l}$ and ${\bar{e}}_{g}$ are the average value of the relative expression levels of lncRNA *l* and PCG *g* across all samples. The cor(*l*, *g*) can be used to effectively evaluate possibility of ceRNAs through measuring expression correlation of the lncRNA‐PCG pair across all samples. The statistical significance of cor(*l*, *g*), termed *P*, was calculated using the significance *P* value of the Pearson correlation coefficient. The Pearson correlation coefficient was adopt by many ceRNA studies and have been proved to be effective for identification of ceRNAs (Paci *et al*., 2014; Wang *et al*., 2015; Xu *et al*., 2015). These existing studies used the absolute expression level of genes, whereas the relative expression levels of genes (log2FC) were considered by previous studies to be able to reduce the influence of heterogeneity among different ESCC patients (Li *et al*., 2014a). Therefore, instead of the absolute expression level, we computed Pearson correlation coefficient by using the relative expression level.

Finally, a lncRNA‐PCG pair sharing miRNAs will be defined as functional ceRNA relationship in ESCC if it meets the following criteria: (a) DEC score(*l*, *g*) > 5; (b) cor(*l*, *g*) > 0 and *P* < 0.05. The above method was applied to all CLIP‐seq‐supported lncRNA‐PCG pairs sharing miRNAs in starBase V2.0 database, and all functional ceRNAs meeting the criteria were identified. The lncRNAs identified in functional lncRNA‐mediated ceRNAs were defined as functional ce‐lncRNAs.

### 2.4 The traditional ceRNA identification methods

Traditionally, a lncRNA‐PCG pair sharing miRNAs will be defined as functional ceRNA relationship based on the following criteria: (a) Expression correlation of lncRNA‐PCG pair; (b) Shared miRNAs; and (c) Differentially expression level of lncRNAs/PCGs. Although most of studies identify ceRNAs based on the three criteria, different combinations of them exist. Therefore, we used six different combinations for fair comparison with our method, including SAM(0.01)+Cor, Limma(0.01)+Cor, SAM(0.05)+Cor, SAM(0.01)+Hyper+Cor, Limma(0.01)+Hype+Cor, and SAM(0.05)+Hype+Cor. Notably, Pearson correlation coefficient (Cor) between a lncRNA‐PCG pair is usually used to identify whether lncRNA‐PCG pair is co‐expressed. All lncRNA‐PCG pairs with Cor > 0 and FDR < 0.05 were identified as candidate ceRNA pairs. The differentially expressed genes are identified using the SAM or Limma method with FDR < 0.01 or 0.05. A hypergeometric test is used to compute significance of shared miRNAs for each possible lncRNA‐PCG pair. All *P* values were subject to FDR correction. For example, Limma(0.01)+Hype+Cor represents that ceRNAs meet significance of expression correlation (+Cor) and share miRNAs (+Hyper) between lncRNA‐PCG pairs, with differentially expression of lncRNAs/PCGs based on Limma FDR < 0.01 (+Limma(0.01)). Limma(0.01)+Cor represents that ceRNAs meet significance of expression correlation between lncRNA‐PCG pairs, with Limma FDR < 0.01, but not use the ‘Shared miRNAs’ criterion with only needing to share at least one miRNA.

### 2.5 Degree and betweenness centrality

The most elementary characteristic of a node is its degree, which represents how many links the node has to other nodes (Barabasi and Oltvai, 2004). Betweenness centrality is a measure of a node's centrality in a network and is equal to the number of shortest paths from each node to all others that pass through this node. It reflects the amount of control that a node exerts over the interactions of other nodes in the network.

### 2.6 Analysis of lncRNA‐related cancer hallmarks

Hanahan and Weinberg (2011) have proposed that cancer cells acquire a number of hallmark biological capabilities during the multistep process of tumor pathogenesis (Hanahan and Weinberg, 2011). We used these hallmarks for analysis of lncRNA‐related cancer hallmarks, including ‘Activating Invasion’, ‘Disrupting Cellular Energetics’, ‘Angiogenesis’, ‘Enabling Replicative Immortality’, ‘Genome Instability’, ‘Resisting Cell Death’, ‘Sustaining proliferative signaling’, ‘Tumor‐Promoting Inflammation’, ‘Evading Growth Suppressors’, and ‘Avoiding Immune Destruction’. To obtain cancer hallmark genes, we firstly corresponded cancer hallmark to the Gene Ontology (GO) terms according to the study of Hnisz *et al*. (2013). Secondly, the genes annotated to these GO terms were downloaded from the databases MsigDB V6.1 (Subramanian *et al*., 2005) and bioMart (Ensembl v91). Thirdly, for each GO term, the union of their related genes obtained from the two databases was used as the annotated genes of the GO term. The result showed that all cancer hallmarks can correspond to 31 GO terms with the annotated genes. Finally, these GO terms were used as proxies for the characteristic hallmark capabilities that are thought to be acquired in cancers.

To test whether ce‐lncRNAs can control broad cancer‐related hallmarks, we investigated ce‐lncRNAs in the context of cancer hallmarks. On the one hand, we mapped all ce‐lncRNA‐related PCGs identified by GloceRNA from the GSE53625 (*n* = 119) and SRP064894 datasets to cancer hallmarks and used hypergeometric test to calculate the enrichment significance of each cancer hallmark GO terms. On the other hand, we explored hallmark functions associated with each ce‐lncRNA. Notably, for each ce‐lncRNA, we used the ce‐lncRNA‐related PCGs from two datasets to identify the enriched hallmark GO terms. The enrichment significance was calculated using hypergeometric test.

### 2.7 Survival analysis

A clear understanding of the alterations in lncRNA expression occurring in cancers will require larger‐scale studies. The GSE53625 (*n* = 119) and GSE53625 (*n* = 60) datasets were used as survival analysis of functional ce‐lncRNAs and ceRNA pairs. The clinical and survival information of patients in the two datasets was provided in Table S1 and Appendix S1. For a lncRNA (or PCG), the relationship between lncRNA (or PCG) expression and prognosis of ESCC patients was explored by Kaplan–Meier analysis (Li et al., 2019). The mean value of gene expression was used as cutoff to classify patients into high‐ and low‐risk groups. The statistical significance was assessed using the log‐rank test by calculating the *P* values. For a ceRNA pair, an average expression of the corresponding lncRNA and PCG was calculated for each patient. Then, we used the average expression level of the ceRNA pair as the ‘pair expression’ to evacuate the association between survival and the ceRNA pair. Similarly, the mean value of ‘pair expression’ was used as cutoff to classify patients into high‐ and low‐risk groups. The statistical significance was assessed using the log‐rank test by calculating the *P* values. The lncRNA, PCG, and the ceRNA pair with *P* < 0.05 were defined as significant. We used the same ‘mean value’ strategy as the cutoff to classify patients into high and low‐risk groups in the GSE53625 (*n* = 119) and GSE53625 (*n* = 60) datasets. All analyses were performed on the r 2.13.2 framework.

### 2.8 Chromatin immunoprecipitation sequencing data analysis

Chromatin immunoprecipitation sequencing (ChIP‐seq) files have been obtained from our previous studies with GEO database (GEO ID: GSE76861 and GSE106563) (Jiang *et al*., 2018b, 2017). H3K27ac ChIP‐seq was sequenced in six ESCC cell lines, including KYSE140, TT, KYSE510, KYSE70, TE5, and TE7. H3K27ac ChIP‐seq reads were mapped using bowtie aligner (v0.12.9) to hg19 human reference genome (Langmead *et al*., 2009). macs (model‐based analysis of ChIP‐seq) (v1.4.2) was used to identify enhancer enrichment regions (Zhang *et al*., 2008). The corresponding wiggle files were generated using read pileups and were normalized using reads per million (rpm) by dividing tag counts by the total number of reads. We converted wiggle files into bigwig files using wigtobigwig tool (http://hgdownload.cse.ucsc.edu/admin/exe/) and visualized them using integrative genomics viewer (http://www.broadinstitute.org/igv/home). rose software was used to identify potential SE regions as ‘python ROSE main.py ‐g hg19 ‐i *******.gff ‐r ******* cas.sort.bam –c ******* input.sort.bam ‐o ******* ‐s 12500 ‐t 2000’ (Hnisz *et al*., 2013). Briefly, H3K27ac peaks that occurred within ±1 kb of transcription start sites were subtracted. rose stitched enhancers within 12.5 kb together. It separated SEs from TEs through ranking H3K27ac signal of them. Finally, a threshold was defined according to the geometric inflection point to distinguish between TE and SE. Both SEs and TEs were assigned to the overlap, proximal, and closest genes to the center of the stitched enhancer. If lncRNAs appeared in the overlap, proximal, or closest genes of SEs or TEs, they were considered as SE/TE‐associated lncRNAs. If SE/TE‐associated lncRNAs belong to ce‐lncRNAs in ESCC, we considered them as SE/TE‐associated ce‐lncRNAs in ESCC.

### 2.9 Identification of transcription factors binding to SEs of ce‐lncRNAs

Identification of transcription factors that were predicted to bind to SEs of lncRNAs was based on motif scanning in SE regions associated with ce‐lncRNAs. More than 3000 DNA binding motifs for 695 transcription factors are compiled from the TRANSFAC database (Matys *et al*., 2006) and MEME suite (Bailey *et al*., 2009), based on the following collections: JASPAR CORE 2014 vertebrates (Mathelier *et al*., 2014), Jolma2013 (Jolma *et al*., 2013), Homeodomains (Berger *et al*., 2008), UniPROBE (Robasky and Bulyk, 2011), and Wei2010 (Wei *et al*., 2010). For each of six ESCC cell line, we obtained the genomic regions of the constituents of SEs associated with ce‐lncRNAs. According to these regions, we extracted their corresponding sequence from hg19 human reference genome using the *getfasta* function of bedtools (v2.25.0) (Quinlan and Hall, 2010) and followed by motif scanning with fimo (Find Individual Motif Occurrences) at a *P* value threshold of 10^−4^ (Grant *et al*., 2011). Transcription factors having at least two significant DNA binding sequence motif instances in the SEs of each ce‐lncRNA were identified. For each of identified transcription factor, we computed unique lncRNAs regulated by it through merging relationships between transcription factors and SE‐associated ce‐lncRNAs for all six ESCC cell lines. All transcription factors were finally ranked according to number of lncRNAs significantly regulated by them.

### 2.10 Gene expression profile for the effects of THZ1 inhibition for lncRNAs and related PCGs

Gene expression profiles for the effects of THZ1 inhibition were performed in our groups. The data can be downloaded from NCBI GEO database (GSE number: GSE76860). The detailed experimental descriptions were provided in our previous published paper (Jiang *et al*., 2017). Briefly, whole‐transcriptome RNA sequencing was performed before/after THZ1 treatment in TE7 and KYSE510 cells using illumina HiSeq 2000. The RNA‐seq results were involved in gene expression level of either THZ1 or DMSO at indicated time points at 2, 4, 6, and 8 h, which were computed using FPKM through mapping reads to human reference genome. We filtered genes according to FPKM, and those active genes with FPKM > 1 were considered in following analyses.

### 2.11 Construction of THZ1‐sensitive ceRNA networks

Firstly, we used gene expression profiles for the effects of THZ1 inhibition to compute fold changes of the expression level for SE/TE‐associated ce‐lncRNAs. If the expression level of SE/TE‐associated ce‐lncRNAs decreased over 1.5‐fold at 12 h compared with DMSO, we defined them as ‘THZ1‐sensitive SE/TE‐ce‐lncRNAs’. A total of 42 unique THZ1‐sensitive SE/TE‐ce‐lncRNAs were identified in TE7 and KYSE510 cells. Secondly, we obtained the 26 shared THZ1‐sensitive SE/TE‐ce‐lncRNAs in both cell lines. Based on these ce‐lncRNAs, we extracted the first neighbor nodes in ESCC ceRNA network, and thus, the related PCGs associated with THZ1‐sensitive SE/TE‐ce‐lncRNAs were obtained. Finally, a subnetwork of ESCC ceRNA network, called THZ1‐sensitive ceRNA networks, was constructed through extracting the subgraph using THZ1‐sensitive SE/TE‐ce‐lncRNAs and their related PCGs. The nodes in the subnetwork are THZ1‐sensitive SE/TE‐ce‐lncRNAs or their related PCGs, and edges are the ceRNA relationships between them.

### 2.12 Cell culture and RNA interference

Cell lines used in this study and related cell culture information has been described previously (Long *et al*., 2018). The KYSE150, KYSE510, and TE3 human esophageal squamous carcinoma cell lines were cultured in Roswell Park Memorial Institute (RPMI) 1640 medium (HYCLONE, Logan, UT, USA). ESCC cell line KYSE450 was cultured in Dulbecco's modification of Eagle's medium Dulbecco (DMEM) medium (Thermo Fisher Scientific, Waltham, MA, USA). All media were supplemented with 10% FBS (Thermo Fisher Scientific), penicillin‐G (100 units·mL^−1^), and streptomycin (100 μg·mL^−1^). Cells were incubated at 37 °C in a humidified atmosphere containing 5% CO_2_.

In functional assays, KYSE150, KYSE450, and TE3 cells were seeded into 6‐well plates or 12‐well plates and cultured for 12–24 h until 70–80% confluence. ESCC cells were transfected with 25 or 50 nm small interfering RNA (siRNA) using DharmaFECT™ Transfection Reagents (Dharmacon, Waltham, MA, USA) or Lipofectamine 3000 (Invitrogen, Carlsbad, CA, USA) according to the manufacturer's instructions. The LINC00094, LINC00338, SNHG10, MFI2‐AS1, and a negative control (NC) siRNAs were synthesized by Dharmacon. The TCF3 and KLF5 siRNAs were synthesized by GenePharma (Suzhou, China). The siRNA target sequence for lncRNAs and two transcription factors' mRNAs is described in Table S4.

### 2.13 RNA extraction and qRT‐PCR

Total RNA from ESCC cells were extracted using TRIzol (Invitrogen) according to the manufacturer's protocol. The purity and concentration of RNA were determined by OD260/280 using a NanoDrop ND‐2000 spectrophotometer (Agilent, Santa Clara, CA, USA), and 1 μg of total RNA was reverse transcribed into cDNA using PrimeScript RT reagent Kit with gDNA Eraser (TaKaRa, Otsu, Japan) in accordance with the manufacturer's instructions. Quantitative real‐time PCR (qRT‐PCR) was performed by SYBR Premix Ex Taq (TaKaRa) using a 7500 Real‐Time PCR System (Applied Biosystems, Waltham, MA, USA). Primers for quantitative real‐time PCR are shown in Table S5. β‐Actin was measured as an internal control and used for normalization. RNA expression was normalized against the relative value from the NC control group. qRT‐PCR was performed in triplicate and repeated at least three times.

### 2.14 Wound healing assay

KYSE150, KYSE450, and TE3 cells were transfected with siRNAs targeting lncRNAs, and then, cells were starved in serum‐free medium for 12 h after being transfected for 36 h. Circles 3 mm in diameter were marked on the bottom of each dish to identify the areas for image capture and ensure that measurements were taken at the same locations. A wound was made by scraping the cell monolayer with a 200‐μL pipette tip. ESCC cells were maintained in RPMI‐1640 medium or DMEM medium with 2.5% FBS. Images were captured at 0 and 36 h using a Leica DMI3000B inverted phase‐contrast microscope (Leica Microsystems GmbH, Wetzlar, Germany). The wound closure rate was calculated from six images, using imagej (National Institutes of Health, Bethesda, MD, USA) analysis. Each experiment was performed in triplicate.

### 2.15 Transwell assay

Transwell assay was performed as described previously (Zhang *et al*., 2018c). KYSE150, KYSE450, and TE3 cells were starved in serum‐free medium for 12 h after being transfected. A total of 5 × 10^4^ cells were plated in medium without serum in the upper well of a transwell chamber of a 24‐well transwell with 8‐μm pores (BD Biosciences, San Jose, CA, USA), placed in a bottom chamber containing medium supplemented with 10% FBS. After 48 h, the membranes were fixed ice‐cold methanol and stained with hematoxylin solution, and migration was quantified by counting 10 random fields under a Leica DMI3000B inverted phase‐contrast microscope (400×). The migration cell numbers were counted with imagej. Each experiment was performed in triplicate.

### 2.16 Colony formation assay

Colony formation assay was performed as described previously (Zeng *et al*., 2017). Briefly, transfected cells were trypsinized and counted with a cell counter (Bio‐Rad, Hercules, CA, USA). Then, cells were plated at a density of 1000 cells per well in 6‐well plates and incubated for 14 days at 37 °C with 5% CO_2_. After washing with 4 °C precooled PBS twice, cultures were fixed with ice‐cold methanol for 15 min and stained with hematoxylin for 30 min. Colonies were photographed by ChemiDoc Touch (Bio‐Rad). Each experiment was performed in triplicate.

### 2.17 Western blotting

ESCC cells were lysed with Laemmli sample buffer (Bio‐Rad), heated for 10 min at 95°C. Western blotting was performed using SDS/PAGE. Proteins were transferred to PVDF membrane (Millipore, Billerica, MA, USA), which were then blocked for 1 h with 5% skim milk in TBST (20 mm Tris, 137 mm NaCl, 0.1% Tween‐20). Membranes were incubated with primary antibody [1 : 1000 anti‐KLF5 (Santa Cruz Biotechnology, Delaware Ave, Santa Cruz, CA, USA; sc‐398470) and anti‐TCF3 (Cell Signaling Technology, Danvers, MA, USA; Cat#4865) and anti‐β‐actin (Santa Cruz Biotechnology; sc‐47778)] overnight in 4 °C. After three washes with TBST, membranes were incubated with secondary HRP‐conjugated antibody [1 : 5000 (mouse; Santa Cruz Biotechnology; sc‐516102) and 1 : 2000 (rabbit; Cell Signaling Technology; cat# 31,460)] for 1 h at room temperature. Signals were detected with ChemiDoc Touch (Bio‐Rad).

### 2.18 Chromatin immunoprecipitation

Chromatin immunoprecipitation analysis was performed as described previously (Jiang *et al*., 2018b). In brief, ESCC cells KYSE150, KYSE510, and TE3 were treated with THZ1 (100 nm, 12 h), and then, 1 × 10^7^ cells were cross‐linked with 1% formaldehyde solution (Thermo Fisher Scientific) and neutralized by 1.25 m glycine. Cross‐linked cells were lysed and sonicated (Covaris E220, Woburn, MA, USA) to release 100–00 bp fragments. Anti‐KLF5 (Santa Cruz Biotechnology; sc‐398470x), anti‐TCF3 (Santa Cruz Biotechnology; sc‐166411x), or normal IgG was added to each sonicated chromatin and incubated at 4 °C overnight. Then, these complexes were conjugated to Dynabeads protein A/G magnetic beads (Invitrogen) for 4 h at 4 °C. After incubation, DNA was eluted from immunoprecipitate complexes, reverse cross‐linked, and purified with QIAquick PCR purification kit (QIAGEN, Hilden, Germany).

The purified DNA was analyzed by real‐time PCR with the use of LINC00094 super‐enhancer‐specific primers. Primers for ChIP‐PCR were shown in Table S5. Relative enrichment was normalized to input. IgG antibody was used as a negative control.

### 2.19 Statistical analysis

Results are analyzed by spss software, 13.0 (SPSS, Chicago, IL, USA) or r 3.1.2 for windows. Where indicated, statistical analysis was performed by calculating means and SD. Graphs about biological experiments were mainly made by graphpad prism 6 (GraphPad, San Diego, CA, USA). Differences between groups were evaluated with the Student's *t*‐test. *P* < 0.05 was considered to be statistically significant. *P < 0.05, **P < 0.01, ***P < 0.001. Graphs about bioinformatics were mainly made by r 3.1.2.

## 3 Result

### 3.1 Genome‐wide identification of ce‐lncRNAs using GloceRNA

To systematically identify functional ce‐lncRNAs, we developed a two‐stage identification method, termed GloceRNA, which integrated miRNA target sequences and gene expression profile information of lncRNAs and PCGs in large‐scale N/T matched samples (see Materials and methods). Our hypothesis is that functional ceRNAs display expression direction consistency in local matched samples and at global gene expression level cross all samples. Briefly, lncRNA‐PCG pairs sharing miRNA target sites were first established through using CLIP‐seq‐supported miRNA‐PCG and miRNA‐lncRNA interactions (Fig. 1A). Next, each lncRNA‐PCG pair sharing miRNAs was tested using two measures DEC score(*l*, *g*) and cor(*l*, *g*) and identified as a functional ceRNA relationship if it meets the following criteria: (a) local regulatory direction consistency of expression at single sample level (DEC score(*l*, *g*) > 5) (Fig. 1A,B); (b) global regulatory direction consistency of expression (cor(*l*, *g*) > 0 and *P* < .0.05) (Fig. 1A,C). Finally, the related lncRNAs in functional ceRNAs were identified as functional ce‐lncRNAs. The GloceRNA method has two advantages. On the one hand, using a new measure DEC score(*l*, *g*), local differential expression consistency between lncRNAs and PCGs can be effectively considered through computing regulatory direction consistency of expression at single N/T matched sample level, which can effectively evaluate possibility of ceRNAs appearing in parts of samples. On the other hand, global expression consistency of a lncRNA‐PCG pair is tested through applying Pearson correlation coefficient to all samples, which can effectively evaluate possibility of ceRNAs through measuring relative expression correlation of the lncRNA‐PCG pair cross all samples. Therefore, our method not only considered regulatory direction consistency of expression at the global level but also mined hidden regulatory direction information of ceRNAs from single and local some N/T matched samples.

**Fig. 2:**
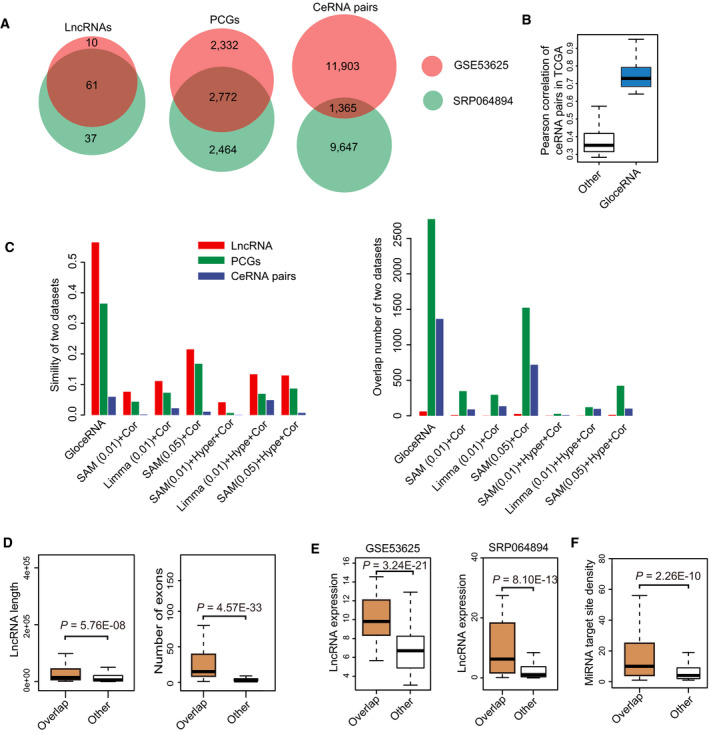
Identification and analysis of functional ce‐lncRNAs in ESCC. (A) Venn diagram showing the overlap of lncRNAs (left), PCGs (middle), and ceRNA pairs (right) between both ESCC datasets (GSE53625 and SRP064894). (B) Box plots of expression correlation of ceRNA pairs in TCGA ESCC samples. The bars represent expression of ceRNA pairs (blue) and all background pairs (write). (C) Comparison of results between our method and other methods. Left panel shows overlap similarity of lncRNAs (red), PCGs (green) and ceRNA pairs (blue). Right panel shows overlap number of lncRNAs (red), PCGs (green), and ceRNA pairs (blue). Traditionally, a lncRNA‐PCG pair sharing miRNAs will be defined as functional ceRNA relationship based on the following criteria: (a) Expression correlation of lncRNA‐PCG pair (Cor); (b) Shared miRNAs (Hyper); and (c) Differentially expression level of lncRNAs/PCGs (SAM or Limma). We used six different combinations of them for fair comparison with our method, including SAM(0.01)+Cor, Limma(0.01)+Cor, SAM(0.05)+Cor, SAM(0.01)+Hyper+Cor, Limma(0.01)+Hype+Cor, and SAM(0.05)+Hype+Cor. Box plots of ce‐lncRNAs are displayed according to (D) Length (left) and number (right) of exons, (E) expression level, and (F) number of miRNA target sites. GSE53625 represents the GSE53625 (*n* = 119) dataset.

Since we have previously characterized several important lncRNAs in ESCC, for which we also generated RNA‐seq data from patient samples (Jiang *et al*., 2018b; Li *et al*., 2017; Xie *et al*., 2018; Zhang *et al*., 2018c), we next applied GloceRNA to this cancer type. Using internal dataset (15 paired tumor and normal samples), 13 268 ceRNA pairs were identified, involving 98 lncRNAs and 5236 PCGs. To evaluate the robustness of this result, we analyzed another large‐scale transcriptomic dataset (119 paired tumor and normal samples). Strikingly, in this independent cohort, 61 out of 71 lncRNAs (85.91%) were significantly shared with our internal dataset (*P* = 0, hypergeometric test, Fig. 2A). The result showed that the ceRNA networks derived from different datasets shared similar lncRNAs. Moreover, we found that the overlaps of PCGs (*P* = 0) and ceRNA pairs (*P* = 0) between the two cohorts were also highly significant statistically (Fig. 2A), highlighting the consistency and robustness of our method. We further found that similarities of nodes and edges were obviously different although the overlaps were highly statistically significant. The overlaps between lncRNAs, as well as PCGs, were much larger than those between ceRNA pairs (Fig. 2A). This suggests that the ceRNA networks derived from different ESCC datasets might more tend to share similar nodes compared with edges. In other words, the nodes in the ESCC ceRNA network may be more conservative than regulatory relationships between nodes in different patients and datasets. In the ESCC tissues of different patients, those lncRNAs, which perform their ceRNA functions, may usually be about the same. Moreover, they tend to regulate the similar terminal target PCGs. However, despite significantly sharing some ceRNA regulatory paths in the different patients, these ce‐lncRNAs may adopt many different regulatory paths to transmit signals and implement the regulation for the same target PCGs.

Next, we compared the GloceRNA with other ceRNA identification methods, including SAM and Limma (Fig. 2C, Fig. S1), and GloceRNA displayed markedly higher consistency and stability compared with either SAM or Limma. To further test the performance of GloceRNA, we analyzed the TCGA ESCC datasets. Because TCGA did not have full matched samples, ceRNAs cannot be identified directly using our method. Alternatively, we computed Pearson correlation of the expression ceRNAs. Indeed, we observed that ceRNA pairs identified by our methods were significantly higher co‐expressed than others (Fig. 2B).

We next focused on the 61 ce‐lncRNAs shared in two datasets. We observed that transcripts for ce‐lncRNAs were longer than other lncRNAs (*P* = 5.76E‐8, Wilcoxon rank‐sum test, Fig. 2D). Moreover, ce‐lncRNAs had more exons per transcript than other lncRNAs (*P* = 4.57E‐33, Wilcoxon rank‐sum test, Fig. 2D). These observations support previous findings that lncRNAs with longer transcripts and a greater number of exons would be expected to have a higher probability of forming sequence structures that harbor miRNA target sites (Wang *et al*., 2015). In addition, these 61 ce‐lncRNAs were expressed higher and contained more miRNA target sites than other lncRNAs (Fig. 2E,F), again consistent with known features of ceRNAs (Wang *et al*., 2015).

### 3.2 The topological network analysis identifies novel functional ce‐lncRNAs in ESCC

**Fig. 3:**
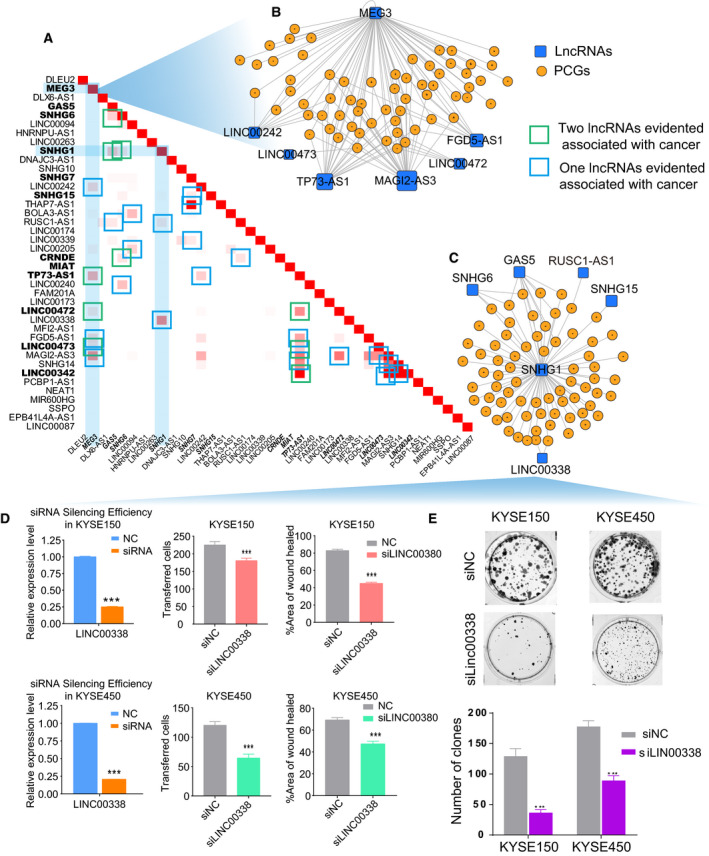
The functional ce‐lncRNAs in the conservative ceRNA network. (A) Similarity between lncRNAs based on the conservative ceRNA network. For a lncRNA‐lncRNA pair, similarity is tested through computing the shared PCGs of two lncRNAs. (B, C) The subnetworks related to MEG3 or SNHG1, respectively. The two subnetworks were extracted from the conservative ceRNA network through considering MEG3 or SNHG1 as the center. Similarities between lncRNAs were tested through computing the shared PCGs of two lncRNAs in the network, which have been provided in (A). (D) Wound healing assay, transwell migration assays, and (E) colony formation assay were performed to determine the effect of LINC00038 on proliferation, migration, and clonogenicity. Mean+‐s.d. are shown, *n* = 3. *P < 0.05, **P < 0.01, ***P < 0.001.

Using internal dataset (15 paired tumor and normal samples), 13 268 ceRNA pairs were identified, involving 98 lncRNAs and 5236 PCGs. We next investigated the 1365 ceRNA pairs shared in two datasets, involving 40 lncRNAs and 1004 PCGs (Right panel, Fig. 2A). To understand this complex regulatory network, we applied the topology theory in biology, wherein biological molecules sharing components within the network are predicted to be more biologically functionally similar. Specifically, we computed the shared PCGs of these lncRNAs in a pair‐wise manner. Importantly, a few known functional lncRNAs in cancer biology were validated by this method (Fig. 3A). For example, the lncRNA MEG3 shared multiple PCGs with six other lncRNAs, of which three (TP73‐AS1, LINC00472 and LINC00473) (Chen *et al*., 2018; Mazor *et al*., 2019; Shen *et al*., 2015) were also confirmed to be of biological significance in cancer (Fig. 3B). On the other hand, we proposed that the functions of poorly characterized lncRNAs may be predicted on the basis of sharing PCGs with known lncRNAs (i.e., guilt‐by association). To address this hypothesis, we tested LINC00338, an uncharacterized lncRNA which shared PCGs with SNHG1 (Fig. 3C). SNHG1 contributes to cell growth and survival in several cancer types, and we also found it connected with other known cancer‐associated lncRNAs, such as GAS5 and SNHG6 (Fig. 3C). To probe the biological function of LINC00338 in ESCC, we examined the effect of LINC00338 knockdown and found that silencing of this lncRNA potently reduced the proliferation, migration and clonogenicity of ESCC cells (Fig. 3D,E). These data demonstrate that our topological network analysis is capable of identifying both known and novel functional ce‐lncRNAs.

### 3.3 Ce‐lncRNAs control broad cancer‐related hallmarks

**Fig. 4:**
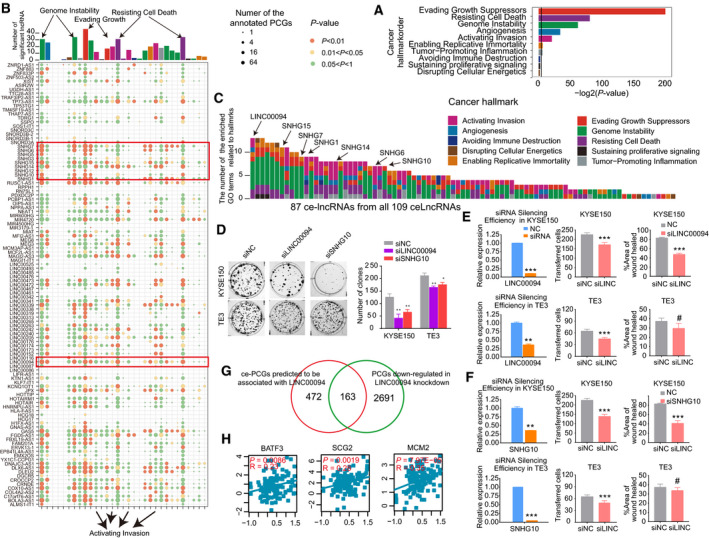
The ceRNA network controls broad cancer‐associated hallmarks. (A) The cancer hallmarks enriched by ce‐lncRNA‐related PCGs in the ceRNA network. The colors of bars correspond to different cancer hallmarks. (B) The summary bubble‐bar plot shows the functional enrichment results of each ce‐lncRNA based on their related PCGs. The top bars show the number of significantly enriched ce‐lncRNAs in each GO term. The ten different colors of bars correspond to ten different cancer hallmarks. The bubble size indicates the number of the annotated ce‐lncRNA‐related PCGs in each term, and different colors correspond to different *P* values. (C) The number of the significantly enriched GO terms for each ce‐lncRNA. Only lncRNAs with at least one enriched GO terms are displayed. These lncRNAs are ranked by number of the GO terms. The colors correspond to different cancer hallmarks. (D) Colony formation assay and (E, F) wound healing assay, and transwell migration assays were performed to determine the effect of LINC00094 and SNHG10 on proliferation, migration, and clonogenicity. Mean ± SD are shown, *n* = 3. *P < 0.05, **P < 0.01, ***P < 0.001, ^#^not significant. (G) Venn diagram showing the overlap between ce‐PCGs predicted to be associated with LINC00094 and PCGs downregulated in LINC00094 knockdown. (H) Gene expression correlation between LINC00094 and several representative ce‐PCGs with cancer hallmark, including BATF3, SCG2, and MCM2. LINC00094 expression level was significantly highly co‐expressed with them based on Pearson correlation coefficient test.

Next, we investigated ce‐lncRNAs in the context of cancer hallmarks. We collected ten cancer hallmarks and their associated genes based on 31 GO terms (Fig. S2A, Appendix S2). Through mapping all ce‐lncRNAs‐related PCGs identified by GloceRNA, we found that seven of ten hallmarks, which corresponded to 18 GO terms, were significantly enriched (*P* < 0.05, hypergeometric test, Fig. 4A, Fig. S2B). The ‘Evading Growth’ hallmark displayed the most significant enrichment, followed by ‘Resisting Cell Death’, ‘Genome Instability’, and ‘Angiogenesis and Activating Invasion’ (hypergeometric test, Fig. 4A). We next explored hallmark functions associated with each ce‐lncRNA through enrichment analysis and revealed that a total of 449 pairs were enriched in the 10 hallmark GO terms (Fig. 4B red and yellow part, Appendix S2). Eighty‐nine out of 109 ce‐lncRNAs were significantly associated with at least cancer hallmark (Fig. 4C). Notably, up to 51 ce‐lncRNAs were significantly enriched in the ‘Cell proliferation’ and ‘Cell_cycle’ terms (Fig. 4B top panel, Fig. S2C).

On the basis of the number of enriched GO terms, LINC00094, a novel lncRNA with unknown functions, was top ranked, and it was enriched in several cancer hallmarks, including ‘Evading Growth’, ‘Resisting Cell Death’, ‘Genome Instability’, ‘Angiogenesis’, and ‘Activating Invasion’ (Fig. 4C). To test this, we silenced this lncRNA and observed that LINC00094 knockdown significantly inhibited proliferation, migration and clonogenicity in ESCC cells (Fig. 4D,E). More importantly, RNA‐seq data showed that the downregulated PCGs upon LINC00094 knockdown significantly overlapped with those predicted by GloceRNA, strongly validating our method (*P* = 7.97E‐05, hypergeometric test, Fig. 4G). Some of LINC00094 target PCGs have well‐known functions in cancer, including BATF3, SCG2, and MCM2. Expectedly, their expression levels were significantly correlated with LINC00094 (*P* = 7.97E‐05, Pearson correlation coefficient test, Fig. 4H).

In addition to LINC00094, we also noted that small nucleolar RNA host genes (SNHGs), including SNHG15, SNHG7, SNHG1, SNHG14, SNHG6, SNHG5, SNHG12, and SNHG10, were significantly enriched to most of hallmarks (Fig. 4C). Moreover, their related PCGs were with high number of annotated genes (Fig. 4B). Interestingly, multiple small nucleolar RNA host genes were recently frequently reported in cancers (Damas *et al*., 2016; Dong *et al*., 2018; Guo *et al*., 2018; Jiang *et al*., 2018a; Shan *et al*., 2018; Sun *et al*., 2017; Xu *et al*., 2018; Zhu *et al*., 2019). In ESCC, we found that knockdown of SNHG10, an uncharacterized lncRNA, reduced proliferation, migration, and clonogenicity in KYSE150 and TE3 cells (Fig. 4D,F). These results suggest that our hallmark enrichment analysis of ce‐lncRNAs may be used to identify additional functional lncRNAs in cancer biology.

### 3.4 Survival analysis of ce‐lncRNAs

**Fig. 5:**
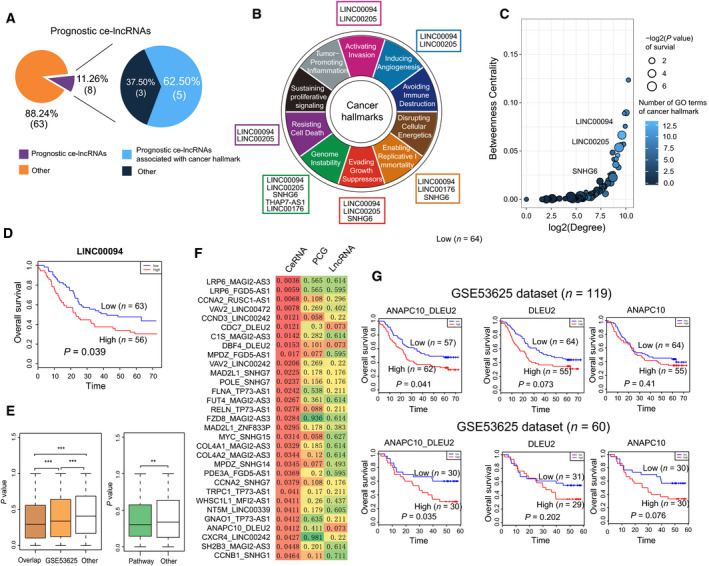
Prognostic analysis of ce‐lncRNAs. (A) The pie charts show the proportion of prognostic ce‐lncRNAs (covered by cancer hallmarks). (B) Diagram of the ten hallmarks of cancer adapted from Hanahan and Weinberg (2011). The prognostic ce‐lncRNAs were assigned to hallmark categories based on GO terms enriched by ce‐lncRNA‐related PCGs. (C) The summary bubble plot showing the relationships between topological feature and number of hallmark GO terms of SE‐associated lncRNAs. X‐ and y‐axis represent degree and betweenness of ce‐lncRNAs in the ceRNA network. The bubble size indicates number of hallmark GO terms. (D) Kaplan–Meier survival curves of patients with ESCC classified into high‐ and low‐risk groups based on the signature of lncRNA LINC00094. (E) Left panel: box plots of the lncRNA‐PCG ceRNA pairs from pairs in the conservative ceRNA network (overlap), pairs identified by GSE53625 (*n* = 119), as well as other random pairs. Right panel: box plots of the lncRNA‐PCG ceRNA pairs annotated to functional pathways. *P* values were calculated using Wilcoxon rank‐sum test. *P < 0.05, **P < 0.01, ***P < 0.001. (F) The lncRNA‐PCGs ceRNA pairs that distinguish ESCC patients better than the corresponding single gene. This means that the ceRNA pair using the log‐rank test with *P* values < 0.05 was identified significant, whereas individual lncRNA and PCG were not significant. Color was related to *P* values. (G) Kaplan–Meier survival curves of ESCC patients the GSE53625 (*n* = 119) and GSE53625 (*n* = 60) datasets that were classified into high‐ and low‐risk groups based on ceRNA pair signature, as well as their corresponding lncRNA and PCG.

An increasing number of studies have suggested that lncRNAs acting as ceRNAs can be powerful predictors of survival in cancer patients (Wang *et al*., 2015; Xu *et al*., 2015). We next explored the relationship between ce‐lncRNA expression and prognosis of ESCC patients by Kaplan–Meier analysis and log‐rank test. Eight of 61 (11.26%) ce‐lncRNAs were identified with *P* < 0.05 (Fig. 5A). Five of them were associated with cancer hallmarks (Fig. 5B). Three ce‐lncRNAs including LINC00094, LINC00205, and SNHG6 exhibited higher degree/betweenness in the ceRNA network and more numbers of hallmarks than most of other ce‐lncRNAs (Fig. 5C). For the lncRNA LINC00094, patients with high lncRNA expression have significantly shorter overall survival (OS) than those with the low expression (Fig. 5D). These lncRNAs were all enriched to ‘Evading Growth’, a hallmark most significantly enriched by ce‐lncRNA network (Figs 4A and 5B). These data suggest that these three lncRNAs might have potential biological significance in ESCC.

Further exhaustive survival analysis was performed on each ceRNA pair (i.e., a pair of lncRNA and PCG) to test their prognostic value. We observed that the ceRNA pairs identified by GloceRNA were more associated with ESCC prognosis than random pairs (*P* = 1.16E‐38, Wilcoxon rank‐sum test), with the ceRNA pairs in the topological ceRNA network being more associated (Fig. 5E, ‘overlap’ in Left panel). Moreover, the ceRNA pairs annotated to functional pathways were more associated with prognosis than others (*P* < 0.01, Wilcoxon rank‐sum test, Fig. 5E, Right panel). Specifically, a total of 31 lncRNA‐PCG pairs were significantly associated with ESCC prognosis (Fig. 5F). As an example for the ceRNA pair of ANAPC10‐DLEU2, patients with high expression have significantly shorter OS than those with the low expression in the cohort of 119 patients (the GSE53625 *n* = 119 dataset) (Fig. 5G, Top panel), which was validated in another independent ESCC cohort (GSE53625 *n* = 60 dataset) (Fig. 5G, Bottom panel). These data indicate that functional ce‐lncRNAs and ceRNA pairs have prognostic value in ESCC.

### 3.5 SEs play key roles in the regulation of ce‐lncRNAs

**Fig. 6:**
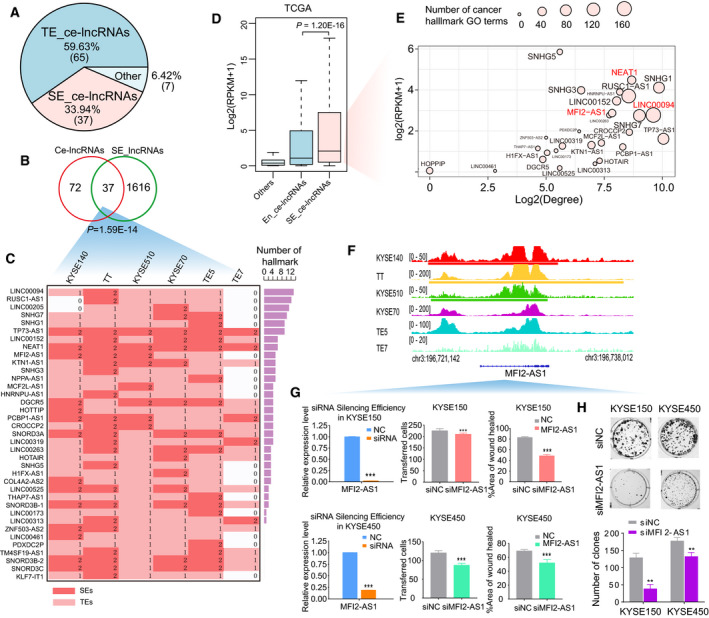
Global overview of SE/TE‐associated ce‐lncRNAs. (A) Pie chart displays SE‐ and TE‐associated ce‐lncRNAs. (B) Venn diagram showing the overlap between ce‐lncRNAs and SE‐associated lncRNAs. SE‐associated lncRNAs are union of lncRNAs associated with SEs appearing in six cell lines. (C) The distribution of SEs/TEs in six cell types about the SE‐associated ce‐lncRNAs. The colored grids represent the TE‐associated (red) or SE‐associated (light red) ce‐lncRNAs involved in each cell type. The ce‐lncRNAs at least appearing in a cell line are listed on the left. The bars on the right show number of GO terms of cancer hallmarks. (D) Box plots of expression (reads per kilobase of exon per million mapped reads, RPKM) from TE‐ and SE‐associated ce‐lncRNAs, as well as other nonenhancer ce‐lncRNAs. The expression of lncRNAs was obtained from TCGA, and all disease samples of ESCC were considered. *P* values was calculated using Wilcoxon rank‐sum test. (E) The summary bubble plot showing the relationships between topological feature, expression, and number of hallmark GO terms of SE‐associated lncRNAs. *X*‐ and *y*‐axis represent degree in the ceRNA network and expression of lncRNAs. The bubble size indicates number of hallmark GO terms. (F) H3K27ac chromatin immunoprecipitation sequencing (ChIP‐seq) binding profiles of representative SE‐associated ce‐lncRNAs in six cell lines. (G) Wound healing assay, transwell migration assays, and (H) colony formation assay were performed to determine the effect of MFI2‐AS1 on proliferation, migration, and clonogenicity. Mean ± SD are shown, *n* = 3. *P < 0.05, **P < 0.01, ***P < 0.001.

Although the biological functions of a few ce‐lncRNAs have been characterized, the upstream regulatory mechanisms of this class of RNAs are largely unknown. Recent studies have shown that a large number of novel noncoding RNAs can be driven by SEs/TEs, which are important for controlling cell identity and cell type‐specific processes (Duan *et al*., 2016; Hnisz *et al*., 2013; Huang *et al*., 2019; Jiang *et al*., 2018b; Miao *et al*., 2018; Peng *et al*., 2019; Wood *et al*., 2018; Xiang *et al*., 2014; Xie *et al*., 2018). To explore the epigenomic mechanisms regulating the expression of our ce‐lncRNAs, we characterized active cis‐regulatory elements in six ESCC cell lines using H3K27ac ChIP‐seq data (Jiang *et al*., 2018b). We identified SEs and TEs using ROSE software (Hnisz *et al*., 2013) and found that that most of ce‐lncRNAs identified by GloceRNA (102/109, 93%) were associated with SEs/TEs in multiple ESCC cell lines (Fig. 6A,C).

Focusing on SE‐associated lncRNAs, we determined that 37 out of 109 (33.94%) ce‐lncRNAs were assigned to SEs (some examples displayed in Fig. S3), exhibiting 3‐fold enrichment than total lncRNAs (*P* = 1.59E‐14, hypergeometric test, Fig. 6B). Expectedly, SE‐associated ce‐lncRNAs were expressed at higher levels than TE‐associated ce‐lncRNAs in TCGA ESCC samples (*P* = 1.20E‐16, Wilcoxon rank‐sum test, Fig. 6D). Moreover, SE‐associated ce‐lncRNAs had higher prognostic value than TE‐associated ce‐lncRNAs (Fig. S4). These data imply that SE‐associated ce‐lncRNAs might be of more biological importance.

We next correlated the expression level, the topological interactive degree, and cancer hallmark analysis of SE‐associated ce‐lncRNAs. Importantly, we observed that SE‐associated ce‐lncRNAs with higher topological degree were strongly associated with expression level and the number of cancer hallmarks enriched (Fig. 6E). For example, LINC00094 had the 3rd strongest topological degree, was enriched in the largest numbers of hallmarks, and was expressed at 9th of all ce‐lncRNAs. The well‐established lncRNA NEAT1, a SE‐associated ce‐lncRNAs, was also top ranked in terms of expression level, the topological degree and cancer hallmark enrichment. We next explored whether we could identify novel functional ce‐lncRNAs by this integrative analysis. We selected a new SE‐associated ce‐lncRNA (MFI2‐AS1), whose SEs appeared in multiple cell lines, was confirmed by us as functionally oncogenic lncRNAs (Fig. 6F–H).

### 3.6 THZ1 inhibits SEs associated ce‐lncRNAs

**Fig. 7:**
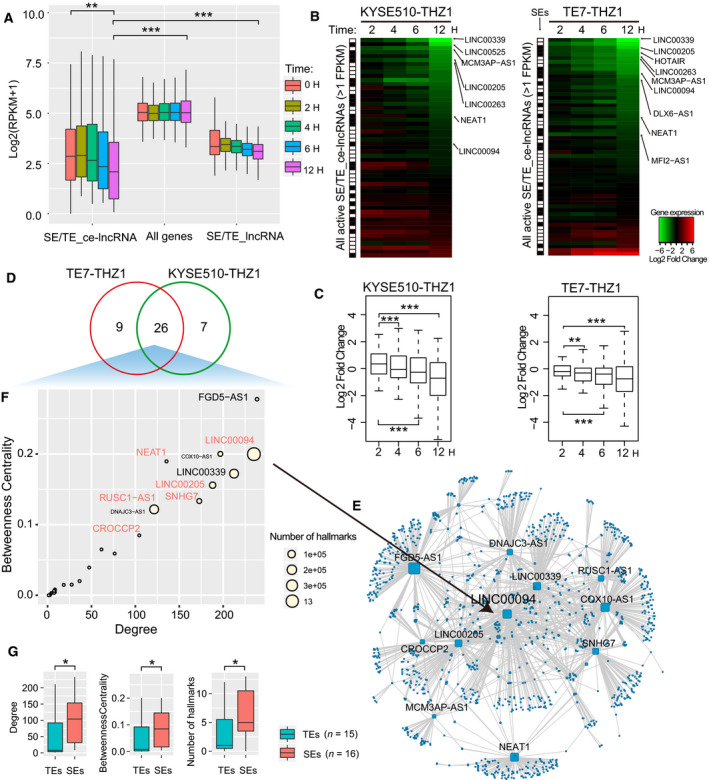
Inhibition of THZ1 for SE/TE‐associated ce‐lncRNAs. (A) Boxplot of expression of enhancer associated ce‐lncRNAs upon either DMSO or THZ1 (50 nm) at indicated time points. (B) Heatmap showing expression changes (log2 fold changes) of all active TE/TE‐associated ce‐lncRNAs upon either DMSO or THZ1 (50 nm) at indicated time points. (C) Box plots of log2 fold changes in global lncRNA expression in KYS510 and TE7 cells treated with either DMSO or THZ1 (50 nm) at indicated time points. (D) Venn diagram showing the overlap between SE/TE‐associated ce‐lncRNAs from KYS510 and TE7 cells which decreased over 1.5‐fold at 12 h. The overlapped lncRNAs were defined as THZ1‐sensitive SE/TE‐ce‐lncRNAs. (E) A THZ1‐sensitive ceRNA network that is constructed using THZ1‐sensitive SE/TE‐ce‐lncRNAs and their related PCGs. (F) The summary bubble plot showing the relationships between topological feature and number of hallmark GO terms of SE‐associated lncRNAs. *X*‐ and *y*‐axis represent degree and betweenness of THZ1‐sensitive SE/TE‐ce‐lncRNAs in the THZ1‐sensitive ceRNA network. The bubble size indicates number of hallmark GO terms. (G) Comparison between SE‐associated and SE‐associated THZ1‐sensitive ce‐lncRNAs, including degrees and betweenness in the THZ1‐sensitive ceRNA network, as well as the number of cancer hallmark GO terms.

To further investigate the regulation dynamics of SEs on these ce‐lncRNAs, we examined the transcriptomic data upon CDK7 inhibition (THZ1), which we have previously shown to preferentially reduce the activity of SEs over TEs (Jiang *et al*., 2017). The effects of THZ1 inhibition for lncRNAs and related PCGs were examined using our previous published gene expression profile (GSE76860) for THZ1 treatment in TE7 and KYSE510 cells (see Materials and methods). The data were involved in gene expression levels associated with either THZ1 or DMSO at indicated time points at 2, 4, 6, and 8 h. We found that although SE/TE‐associated lncRNAs and all background PCGs did not display significant downregulation by THZ1, THZ1 resulted in global downregulation of SE/TE‐associated ce‐lncRNAs at 12 h relative to 0 h (Fig. 7A, Fig. S5). We observed that about half of SE/TE‐associated ce‐LncRNAs were downregulated by THZ1 at 12 h (Fig. 7B,C). We termed this group (SE/TE‐associated ce‐lncRNAs which decreased over 1.5‐fold at 12 h) as ‘THZ1‐sensitive SE/TE‐ce‐lncRNAs’, which comprised 42 ce‐lncRNAs and 26 of them were shared in both cell lines (Fig. 7D).

Focusing on these 26 ce‐lncRNAs, we found that their paired PCGs were also highly sensitive to THZ1 treatment (Fig. S6). We next similarly constructed a THZ1‐sensitive SE/TE‐ceRNA topological network (Fig. 7E) and computed degrees and betweenness of the network for each ce‐lncRNAs. Betweenness is equal to the number of shortest paths from a node to all others that pass through this node, which reflects the ability of control that a node exerts in the network. SE‐associated lncRNAs displayed significantly higher topological importance (degrees and betweenness) than TE‐associated lncRNAs (Fig. 7F,G). Moreover, they regulated significantly more cancer hallmark pathways than TE‐associated lncRNAs (Fig. 7F,G). Some of these SE‐associated lncRNAs including LINC00094, LINC00205, and RUSC1‐AS1, were shown in Fig. 6E.

### 3.7 KLF5 and TCF3 regulated LINC00094 through binding to its SE regions

**Fig. 8:**
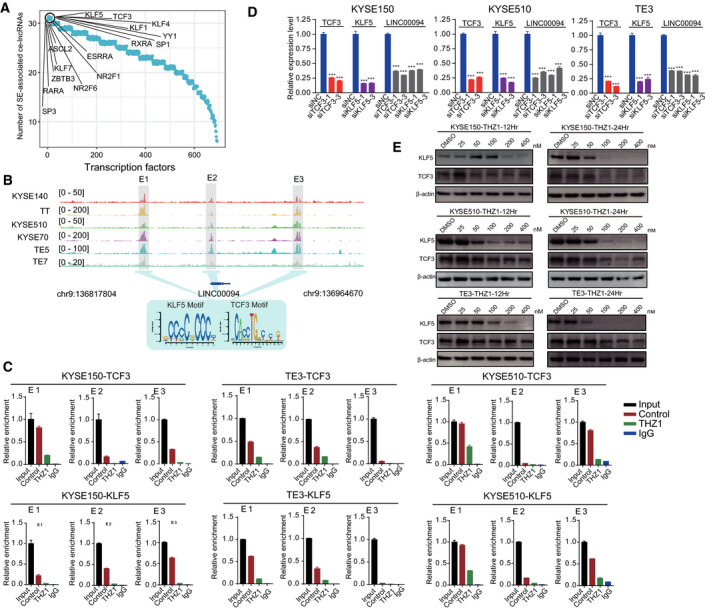
KLF5 and TCF3 bind to SE regions for regulation of LINC00094. (A) The ranked transcription factors according to number of SE‐associated ce‐lncRNAs significantly regulated by transcription factors. (B) H3K27ac ChIP‐seq signals at the LINC00094 locus in six ESCC cell lines. Three constituent enhancers (E1, E2, and E3) within the SE were labeled in grey shadings. TCF3 and KLF5 motif occupy at E1, E2, and E3 enhancer loci. (C) ChIP‐qPCR experiments measuring TCF3 and KLF5 binding enrichment on the LINC00094 SEs segments (divided into enhancer 1, E1; enhancer 2, E2 and enhancer 3, E3) upon treating with THZ1 (100 nm,12 h). Two pairs of primers were designed for each SE segments, which has the better enrichment was finally selected. (D) Relative RNA expression of LINC00094 upon knockdown of TCF3 or KLF5 in KYSE150, KYSE150, and TE3 cells. (E) Western blotting analysis of KLF5 and TCF3 expression in KYSE150, KYSE510 and TE3 cells which were treated with either THZ1 or DMSO at indicated time points and indicated concentrations. Bars of D represent mean ± SD of three experimental replicates. *P < 0.05, **P < 0.01, ***P < 0.001. *P* values were determined using *t*‐test.

Master transcription factors play key roles in regulating the activity of SEs. To identify such transcription factors responsible for the regulation of SE‐associated ce‐lncRNAs, we analyzed the frequency of TF binding motifs within SE regions associated with ce‐lncRNAs via fimo software (Grant *et al*., 2011) from the TRANSFAC database (Matys *et al*., 2006) and MEME suite (Bailey *et al*., 2009). We ranked transcription factors according to number of SE‐associated ce‐lncRNAs significantly regulated by them. 16 transcription factors that regulated most numbers of SE‐associated ce‐lncRNAs were identified (Fig. 8A).

Next, because of the functional importance of LINC00094 for ESCC, we focused on this ce‐lncRNAs to validate the motif analysis results, which predicted the binding of TCF3 and KLF5 to SEs (E1, E2, and E3) of LINC00094 (Fig. 8B). To validate this, ChIP‐qPCR was performed and their enrichment was confirmed at all three SE regions (E1, E2, and E3) (Fig. 8C). Furthermore, we confirmed that THZ1 can inhibit the interaction of TCF3 and KLF5 with the SEs (E1, E2, and E3) (Fig. 8C). More importantly, knockdown of TCF3 or KLF5 significantly downregulated expression of LINC00094 (Fig. 8D, Fig. S7). We also observed decreased expression of TCF3 and KLF5 in a dose‐dependent manner upon THZ1 treatment (Fig. 8E). To further explore the specific mechanism by which signaling pathway this TF‐lncRNA axis regulates, we extracted LINC00094‐related PCGs identified by GloceRNA and annotated these PCGs to KEGG pathways using the iSubpathwayMiner software package (Li *et al*., 2013, 2009). Then, the pathways significantly enriched by LINC00094‐related PCGs were identified using hypergeometric test with FDR corrected *P* < 0.05. We found that signaling pathways and cancer pathways were significantly enriched, including ‘PI3K‐Akt signaling pathway’, ‘Pathways in cancer’, ‘Cell cycle’, and ‘ErbB signaling pathway’. In these pathways, ‘PI3K‐Akt signaling pathway’ contained many LINC00094‐related PCGs (Fig. S8A). Notably, LINC00094 regulated 19 PCGs in the pathway (Fig. S8B). Especially, we found that the core nodes within the ‘PI3K‐Akt signaling pathway’ such as PIK3CA and AKT3 can be regulated by LINC00094 (Fig. S8C). These data demonstrate that TCF3 and KLF5 occupy the SEs of LINC00094, thereby activating its transcription and related downstream signaling pathways in ESCC cells.

## 4 Discussion

Esophageal squamous cell carcinoma is the predominant histological type of esophageal cancer and is considered one of the most common and leading aggressive malignancies with poor prognosis (Jemal *et al*., 2011). In China, over 90% of the cases of esophageal cancer are ESCC, which is the fourth most prevalent cancer of the country (Yang *et al*., 2005; Zhao *et al*., 2010). Recently, researchers have determined the genomic landscape of ESCC and identified a number of driver events (Agrawal *et al*., 2012; Gao *et al*., 2014; Lin *et al*., 2014; Song *et al*., 2014). However, genetic alterations of drug targets are infrequent in patients with ESCC (Agrawal *et al*., 2012; Gao *et al*., 2014; Lin *et al*., 2014; Song *et al*., 2014). Clearly, alternative molecular approaches are needed to further elucidate the pathogenesis of ESCC for developing more innovative and effective regimens. It has now become widely accepted that mammalian genomes encode numerous lncRNAs. Nonetheless, the functional roles of most of these transcripts remain obscure and their upstream/downstream regulatory mechanisms are largely unknown. To systematically pinpoint functional lncRNAs involved in ESCC pathogenesis, we constructed a putative lncRNA‐mediated ceRNA network by integrating lncRNA and PCG expression based on high‐throughput RNA sequencing and microarray data. Based on bioinformatic and experimental approaches, we identified many known and novel functional ce‐lncRNAs and found that most of them acted as a ceRNAs to regulate the expression of broad cancer‐related hallmark genes in ESCC. Interestingly, these lncRNAs acting as ceRNAs were significantly regulated by enhancers, especially SEs. Ce‐lncRNAs have recently been observed to be regulated by SEs. However, ce‐lncRNAs targeted by SEs have not been discovered thus far in ESCC, and the regulation of SEs on ce‐lncRNAs has not been studied.

MiRNAs can mediate ceRNA interaction. If sample matched miRNA, lncRNA, and PCG expression profiles are available, expression correlation of the lncRNA‐miRNA‐PCG triplet can be calculated. Especially, Paci *et al*. developed an effective measure, called sensitivity correlation, to calculate the difference between Pearson and partial correlation coefficients for identification of ceRNAs. However, in the ESCC study, we did not measure expression correlation of the lncRNA‐miRNA‐PCG triplet because our miRNA expression profiles are unavailable. We also found that for many diseases, it is difficult to obtain the sample matched lncRNA, miRNA, and PCG expression profiles. Instead, we focus on prediction of functional ce‐lncRNAs using N/T matched samples. The functional ce‐lncRNAs are predicted using GloceRNA based on merging global and local expression associated with ceRNAs. Especially, using a new measure dec*_i_*(*l*, *g*), expression direction consistency between lncRNAs and PCGs can be effectively considered at single sample level. Suppose that a lncRNA‐PCG ceRNA pair is true. Then, when the expression level of lncRNA in the pair increases in the tumor sample of a patient compared with normal sample, expression of the corresponding PCG should also tend to increase. Therefore, for a pair of N/T samples from the same patient, a ceRNA pair usually displays consistently upregulated (or downregulated) in expression direction. We used dec*_i_*(*l*, *g*) to measure consistency at single sample level, which displayed hidden regulatory direction information of ceRNAs from single N/T matched samples. Based on dec*_i_*(*l*, *g*), we further counted sample number of up/downregulated differential expression consistency across all samples, defined as DEC score, for obtaining local regulatory direction consistency. DEC score can evaluate possibility of ceRNAs significantly appearing in parts of samples through testing times of consistency at single sample level across all samples. We demonstrated that our methods robustly predicted ce‐lncRNAs in multiple ESCC datasets, and the predicted ce‐lncRNAs strongly regulated cancer hallmarks. Moreover, we experimentally validated that some new ce‐lncRNAs predicted by GloceRNA were highly associated with oncogenic functions of ESCC, including LINC00094, LINC00338, SNHG10 and MFI2‐AS1. Especially, a SE‐associated ce‐lncRNA, LINC00094, can promote ESCC cancer cell growth through being activated by TFs binding to SEs. Taken together, if the N/T matched data are available, GloceRNA can provide some useful predictions through effectively using N/T matched samples. GloceRNA thus has potential to complement the existing ceRNA identification methods, as the effective use of N/T matched data and focusing on functional ce‐lncRNAs in ESCC.

We found that most of them significantly regulated the expression of cancer‐related hallmark genes. These ce‐lncRNAs were significantly regulated by enhancers, especially SEs. Landscape analyses for lncRNAs further identified SE‐associated functional ce‐lncRNAs in ESCC, such as HOTAIR, XIST, SNHG5, and LINC00094. THZ1, a specific CDK7 inhibitor, can result in global transcriptional downregulation of SE‐associated ce‐lncRNAs. We further demonstrate that a SE‐associated ce‐lncRNA, LINC00094 can be activated by transcription factors TCF3 and KLF5 through binding to SE regions and promoted ESCC cancer cell growth. THZ1 downregulated expression of LINC00094 through inhibiting TCF3 and KLF5.Our data demonstrated the important roles of SE‐associated ce‐lncRNAs in ESCC oncogenesis and might serve as targets for ESCC diagnosis and therapy.

Efforts to interpret the functional consequences of SEs have mainly focused on the regulation of PCGs, although in a few cases lncRNA regulation was studied. Recent report demonstrated that master transcription factors TP63 and SOX2 promote SCC tumorigenesis such as ESCC through lineage specifically regulating a lncRNA mediated by SEs. We defined a new class of lncRNA, SE‐associated ce‐lncRNA, and performed a thorough investigation of its functional relevance in ESCC cancer cells. Some SE‐associated ce‐lncRNAs with high degree/betweenness were highly associated with cancer hallmarks, including lncRNAs reported in cancer (e.g., NEAT1, HOTAIR, XIST, and SNHG5). Two novel SE‐associated ce‐lncRNAs (LINC00094 and MFI2‐AS1) was identified and validated by us as functionally oncogenic lncRNAs. Our previous studies showed that the unbiased high‐throughput small‐molecule inhibitor screening discover a highly potent anti‐ESCC compound, THZ1, a specific CDK7 inhibitor. Targeting SE‐associated coding gene activation by THZ1 shows powerful antineoplastic properties against ESCC cells (Jiang *et al*., 2017). Furthermore, we found that THZ1 resulted in global downregulation of SE/TE‐ce‐lncRNAs. Furthermore, 26 THZ1‐sensitive SE/TE‐ce‐lncRNAs in both cell lines were identified by us and the related THZ1‐sensitive ceRNA network was extracted. In the network, SE‐associated lncRNAs displayed significantly higher topological importance than TE‐associated lncRNAs. Moreover, they significantly regulated more cancer hallmark pathways than TE‐associated lncRNAs, such as LINC00094, LINC00205, and RUSC1‐AS1. Our findings support recent studies suggesting that SEs can function as important regulators of lncRNAs. SEs play important roles by ce‐lncRNAs.

In process of analysis, we found an important functional ce‐lncRNA, LINC00094. The enrichment analysis showed that the SE‐associated lncRNA was closely related to more than number of cancer hallmarks than other lncRNAs (Fig. 4). LINC00094 parted in core cancer hallmark of ESCC ceRNAs such as ‘Evading Growth’ and ‘Genome Instability’, and its overexpression was highly associated with poor clinical outcome in ESCC patients (Fig. 5D). In all eight significant prognostic ce‐lncRNAs, LINC00094 was with highest degree, betweenness in the ceRNA network and related to most numbers of hallmarks (Fig. 5C). LINC00094 was strongly inhibited by THZ1 and located at the center of the THZ1‐sensitive ceRNA network (Fig. 7E,F). Krüppel‐like transcription factors (KLF) play important roles in development and cancer. KLF4 is a master transcription factor for maintaining the pluripotency of embryonic stem cells (Takahashi and Yamanaka, 2006). It has been reported that KLF5 is highly expressed in multiple cancer types and promotes cancer cell proliferation, migration and survival (Ben‐Porath *et al*., 2008; Chia *et al*., 2015; Jia *et al*., 2016; Nandan *et al*., 2008; Qin *et al*., 2015; Zhang *et al*., 2018b). Especially, KLF5 activates cell identity genes and cancer genes in squamous cell carcinomas (Nakaya *et al*., 2014). KLF5 is also able to occupy the lncRNA RP1 promoter to enhance RP1 expression, which plays an oncogenic role in breast cancer (Jia *et al*., 2019). All the evidence indicates the importance of KLF5 activation in human cancer. We confirmed that transcription factors TCF3 and KLF5 occupied the SE constituents of LINC00094, thereby activating its transcription in ESCC cells. THZ1 decreased expression of TCF3 and KLF5 and inhibited the occupancy of TCF3 and KLF5 (Fig. 8). These results demonstrate that TCF3 and KLF5 can occupy the SEs of LINC00094, thereby activating its transcription in ESCC cells. THZ1 downregulated expression of LINC00094 through inhibiting TCF3 and KLF5.

GloceRNA successfully predicted many ce‐lncRNAs and experimentally validated some new functional ce‐lncRNAs. However, our study has also some limitations. For example, we integrated large‐scale CLIP‐seq (HITS‐CLIP, PAR‐CLIP, iCLIP, CLASH) from the starBase database to obtain enough experimental miRNA‐lncRNA and miRNA‐mRNA interactions. Although these ‘big’ data provided the comprehensive high‐quality information, the datasets used were based on different biological sources such as patients, cell lines, and some did not came from squamous cells and cancer cells. With the accumulation of esophageal squamous cell data, use of cell type‐specific data would be helpful for more accurately identifying ceRNAs and ce‐lncRNAs. Furthermore, there is still much room for improvement in the usability and stability of GloceRNA. For example, although GloceRNA displayed higher stability for identification of ceRNA pairs compared with other state‐of‐the‐art methods, there is still much room for improvement in the stability of ceRNA pairs. Also, the current version of GloceRNA must input the N/T matched data. Therefore, GloceRNA was still unavailable for input of data with nonmatched samples. Some ‘single sample’ strategies of expression profiling analysis may be useful for improving the ability of our method to identify ceRNAs in nonmatched data in the future (Li *et al*., 2020; Liu *et al*., 2017, 2016). In addition, in the current version of GloceRNA, the cutoff of DEC score needs to be manually set and adjusted. The different flexible strategies for setting the cutoff of DEC score, as well as automatic parameter adjustment, would facilitate the identification of functional ce‐lncRNAs. The current strategy for setting the cutoff in the paper is simple and intuitive, and setting the same cutoff in two dataset can also penalize the dataset with small sample size, in which the higher proportion of samples need to meet regulatory direction consistency. We think that other strategies for setting cutoffs may also be effective. For example, the different cutoffs can be selected such as setting the cutoffs according to proportion of samples meeting regulatory direction consistency. With advances in our identification strategy and the accumulation of genomic/transcriptomic profiling data, performance of GloceRNA would continue to improve.

## 5 Conclusion

In summary, we focus on prediction of functional ce‐lncRNAs using N/T matched samples. We developed the GloceRNA method for identification of functional ce‐lncRNAs based on merging global and local regulatory direction consistency of expression associated with ceRNAs. The ce‐lncRNAs unique to squamous cell carcinomas have not been studied extensively. GloceRNA identified many known and novel functional ce‐lncRNAs which regulated the expression of a large number of cancer hallmark genes. Interestingly, we identified novel SE‐associated ce‐lncRNAs in ESCC. Among them, we identified a SE mediated mechanism for the upregulation of a novel oncogenic lncRNA, LINC00094, in ESCC. Considering this gene's ESCC‐specific nature, its association with poor patient survival, and its oncogenic functions, LINC00094 represents a potential biomarker and/or therapeutic target in this group of deadly cancers.

## Conflict of interest

The authors declare no conflict of interest.

## Author contributions

E‐ML, Q‐YW, L‐YX, D‐CL, and C‐QL conceived and devised the study. Q‐YW and LP designed experiments and analysis. LP, Q‐YW, YC, L‐DL, J‐XC, ML, Y‐YL, F‐CQ, Y‐XZ, and FW performed the experiments. Q‐YW, J‐XC, ML, Y‐YL, F‐CQ, Y‐XZ, FW, and C‐QL performed bioinformatics and statistical analysis. Q‐YW, LP, YC, L‐DL, J‐XC, ML, Y‐YL, F‐CQ, Y‐XZ, and FW analyzed the data. Q‐YW, LP, E‐ML, L‐YX, D‐CL, J‐XC, and C‐QL wrote the manuscript.

## Supporting information


**Fig. S1.** Identification ce‐lncRNAs in ESCC. Venn diagram showing the overlap of lncRNAs (left), PCGs (middle) and ceRNA pairs (right) between both ESCC datasets (GSE53625 (n=119) and SRP064894). Traditionally, a lncRNA‐PCG pair sharing miRNAs will be defined as functional ceRNA relationship based on the following criteria: (1) Expression correlation of lncRNA‐PCG pair (Cor); (2) Shared miRNAs (Hyper); (3) Differentially expression level of lncRNAs/PCGs (SAM or Limma). We used six different combinations of them to identify ceRNAs, including (A) SAM(0.01)+Cor. (B) Limma(0.01)+Cor. (C) SAM(0.05)+Cor. (D) SAM(0.01)+Hyper+Cor. (E) Limma(0.01)+Hype+Cor. (F) SAM(0.05)+Hype+Cor.

**Fig. S2.** The ceRNA network controls broad cancer associated hallmarks. (A) The cancer hallmarks corresponding to GO terms. The colors corresponds to different cancer hallmarks. (B) The cancer hallmarks related GO terms enriched by ce‐lncRNA‐related PCGs in the ceRNA network. The colors of bars corresponds to different cancer hallmarks. (C) Number of significantly enriched ce‐lncRNAs for each cancer hallmark.

**Fig. S3.** H3K27ac ChIP‐seq signals at the SE‐associated lncRNA locus in six ESCC cell lines.

**Fig. S4.** Box plots of prognostic value associated with the SE‐associated ce‐lncRNAs, TE‐associated ce‐lncRNAs, as well as other random pairs.

**Fig. S5.** Inhibition of THZ1 for SE/TE‐associated ce‐lncRNAs. (A) Boxplot of expression of SE/TE‐associated ce‐lncRNAs upon either DMSO or THZ1 (50nM) at indicated time points in KYSE510 cells. SE/TE‐associated ce‐lncRNAs were identified in KYSE510 or in all other five cell lines. (B) Boxplot of expression of SE/TE‐associated ce‐lncRNAs upon either DMSO or THZ1 (50nM) at indicated time points, which involved in KYSE510 or TE7 cell lines. * *P* < 0.05, ** *P* < 0.01, *** *P* < 0.001. *P* values were determined using Wilcoxon rank‐sum test.

**Fig. S6.** Box plots of log2 fold changes in expression of lncRNA associated ce‐PCGs in KYS510 and TE7 cells treated with either DMSO or THZ1 (50nM) at indicated time points. * *P* < 0.05, ** *P* < 0.01, *** *P* < 0.001. *P* values were determined using Wilcoxon rank‐sum test.

**Fig. S7.** Western blotting detection for the expression of KLF5 and TCF3 in three ESCC cell lines (KYSE150, KYSE510 and TE3) upon silencing of KLF5 and TCF3 by using different siRNA.

**Fig. S8.** The downstream pathway analysis of LINC00094. (A) The pathways significantly enriched by LINC00094‐related PCGs in the ceRNA network. Enrichment significance was performed by the iSubpathwayMiner software package using hypergeometric test. The pathways with FDR corrected *P* < 0.05 were considered as significant. Number of (*) represents the annotated gene number in the corresponding pathway. (B) LINC00094‐related PCGs that were annotated to the ‘PI3K‐Akt signaling’ pathways. (C) The ‘PI3K‐Akt signaling’ pathways where LINC00094‐related PCGs were annotated. The genes (rectangular nodes) mapped by LINC00094‐related PCGs were shown with red node labels and borders.

**Table S1.** Clinical and pathological characteristics of patients in four datasets for genome‐wide gene expression profiles of ESCC.
**Table S2.** An example of calculating local regulatory direction consistency of a potential lncRNA‐PCG ceRNA pair.
**Table S3.** The overlap and similarity of ceRNA pairs and ce‐lncRNAs identified in two ESCC datasets.
**Table S4.** siRNA target sequences.
**Table S5.** Primers used in this study.

**Appendix S1.** The clinical and pathological characteristics of patients in ESCC datasets.

**Appendix S2.** Cancer hallmarks and their associated genes based on 31 GO terms.

## Data Availability

The datasets used and analyzed during the current study are available from the corresponding author on reasonable request.
