# Supplementary figures and images for: Characterization of super‐enhancer‐associated functional lncRNAs acting as ceRNAs in ESCC
Source: Mol Oncol. 2020 Jun 20;14(9):2203–30. doi: 10.1002/1878-0261.12726 (PMC7463357; doi:10.1002/1878-0261.12726)

**A** SAM (0.01)+Cor

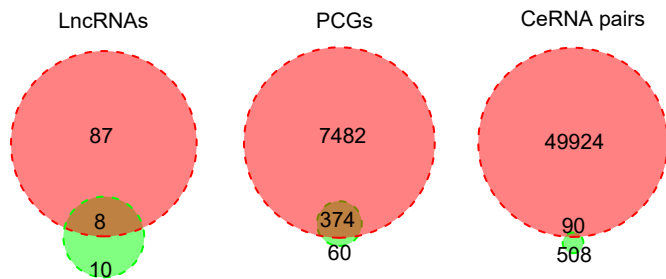

**D** SAM(0.01)+Hyper+Cor

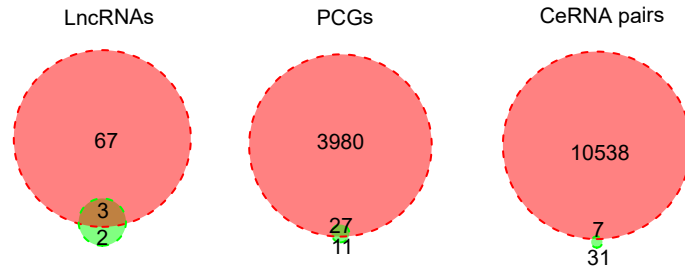

**B** Limma (0.01)+Cor

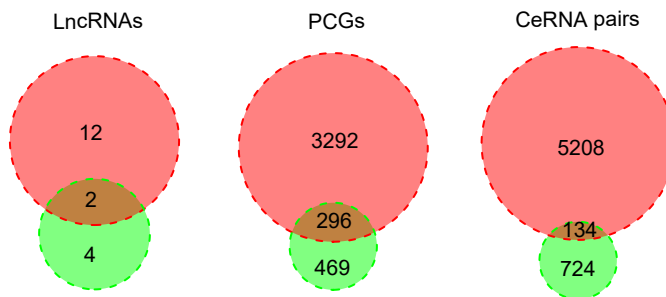

**E** Limma (0.01)+Hype+Cor

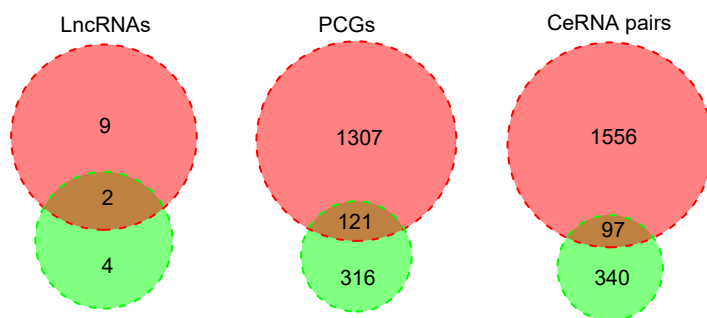

**C** SAM(0.05)+Cor

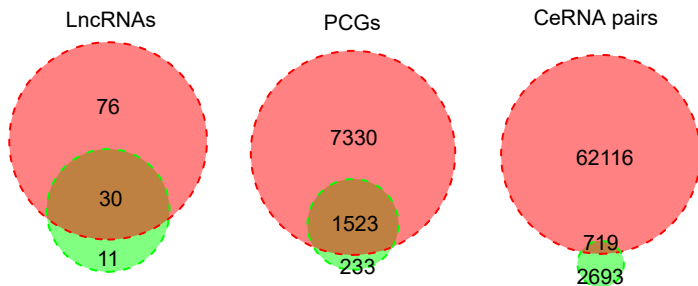

**F** SAM(0.05)+Hype+Cor

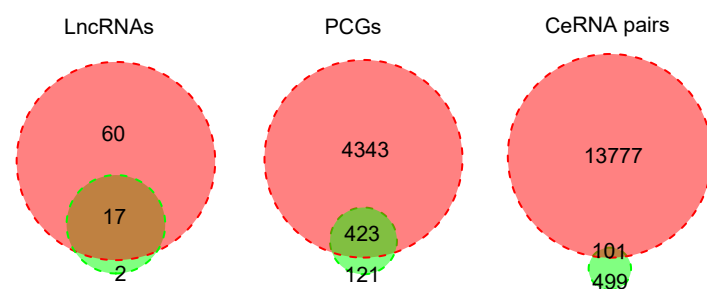

Supplement: Supplementary file 1 — Fig. S1. Identification ce‐lncRNAs in ESCC. Venn diagram showing the overlap of lncRNAs (left), PCGs (middle) and ceRNA pairs (right) between both ESCC datasets (GSE53625 (n=119) and SRP064894). Traditionally, a lncRNA‐PCG pair sharing miRNAs will be defined as functional ceRNA relationship based on the following criteria: (1) Expression correlation of lncRNA‐PCG pair (Cor); (2) Shared miRNAs (Hyper); (3) Differentially expression level of lncRNAs/PCGs (SAM or Limma). We used six different combinations of them to identify ceRNAs, including (A) SAM(0.01)+Cor. (B) Limma(0.01)+Cor. (C) SAM(0.05)+Cor. (D) SAM(0.01)+Hyper+Cor. (E) Limma(0.01)+Hype+Cor. (F) SAM(0.05)+Hype+Cor. [file MOL2-14-2203-s001.pdf]

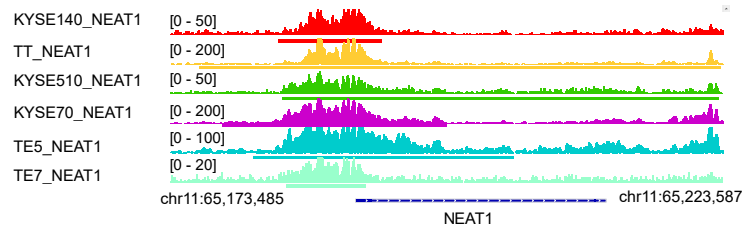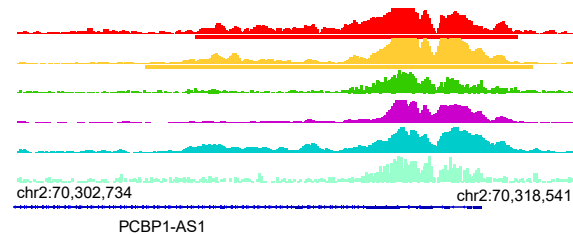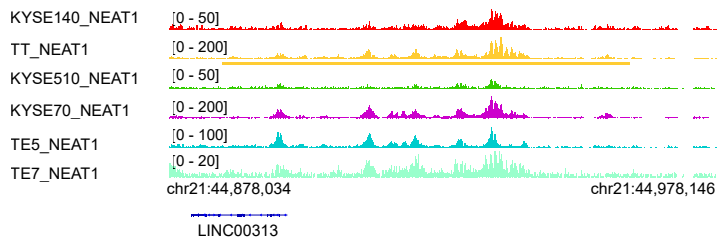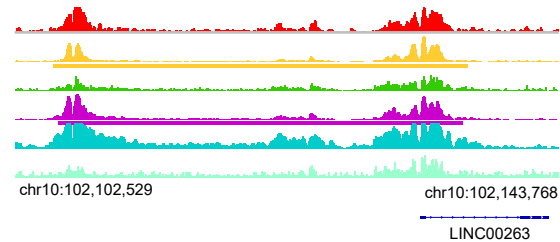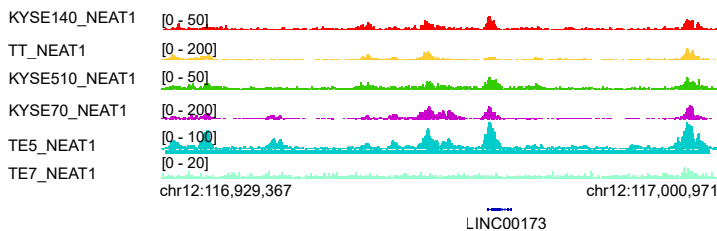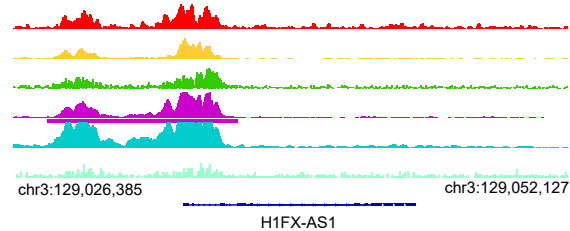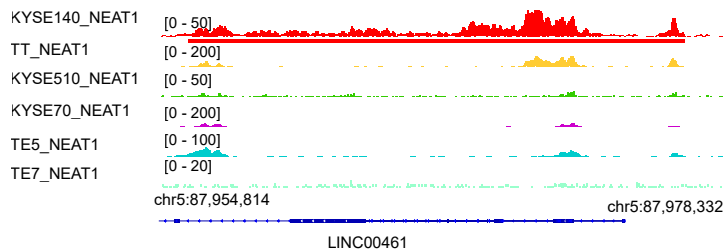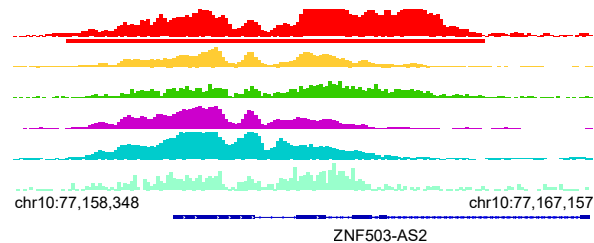

Supplement: Supplementary file 3 — Fig. S3. H3K27ac ChIP‐seq signals at the SE‐associated lncRNA locus in six ESCC cell lines. [file MOL2-14-2203-s003.pdf]

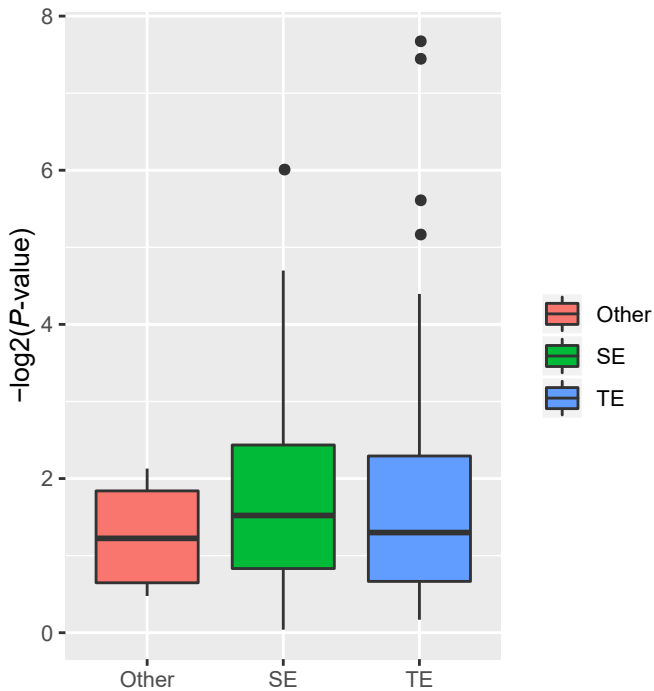

Supplement: Supplementary file 4 — Fig. S4. Box plots of prognostic value associated with the SE‐associated ce‐lncRNAs, TE‐associated ce‐lncRNAs, as well as other random pairs. [file MOL2-14-2203-s004.pdf]

A

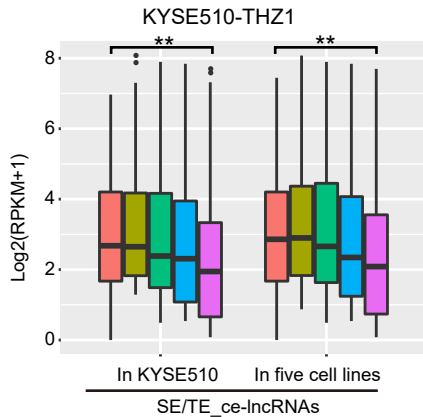

B

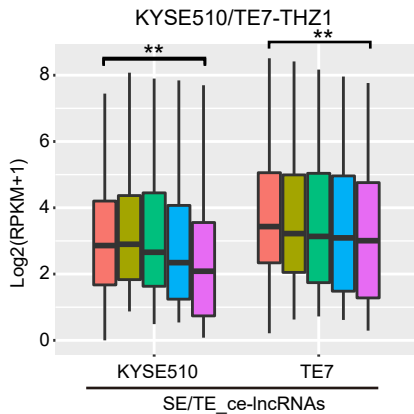

Supplement: Supplementary file 5 — Fig. S5. Inhibition of THZ1 for SE/TE‐associated ce‐lncRNAs. (A) Boxplot of expression of SE/TE‐associated ce‐lncRNAs upon either DMSO or THZ1 (50nM) at indicated time points in KYSE510 cells. SE/TE‐associated ce‐lncRNAs were identified in KYSE510 or in all other five cell lines. (B) Boxplot of expression of SE/TE‐associated ce‐lncRNAs upon either DMSO or THZ1 (50nM) at indicated time points, which involved in KYSE510 or TE7 cell lines. * P < 0.05, ** P < 0.01, *** P < 0.001. P values were determined using Wilcoxon rank‐sum test. [file MOL2-14-2203-s005.pdf]

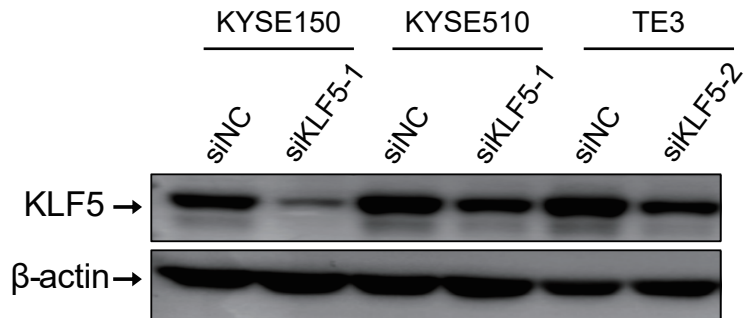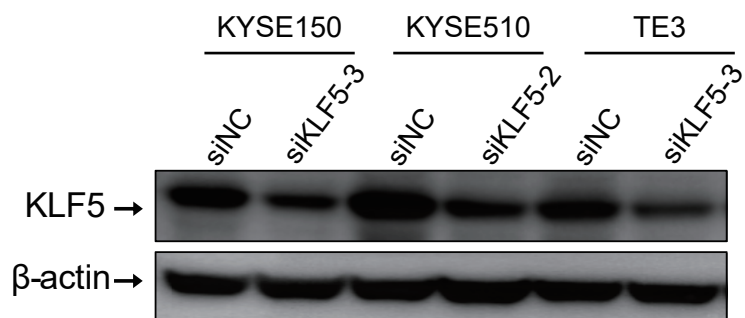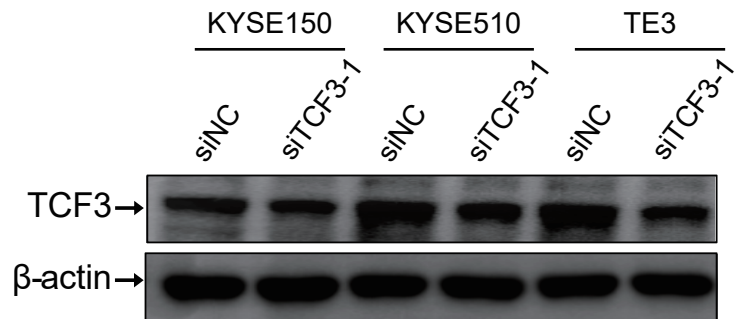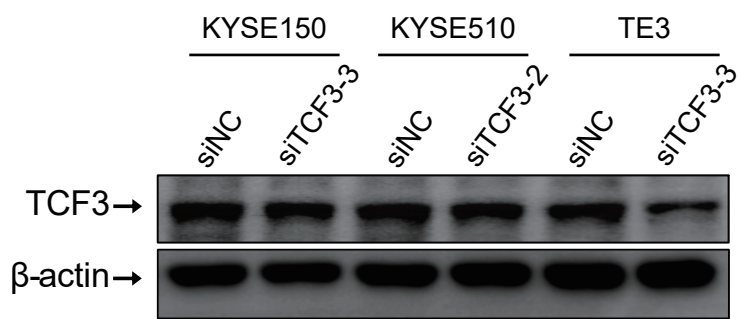

Supplement: Supplementary file 7 — Fig. S7. Western blotting detection for the expression of KLF5 and TCF3 in three ESCC cell lines (KYSE150, KYSE510 and TE3) upon silencing of KLF5 and TCF3 by using different siRNA. [file MOL2-14-2203-s007.pdf]
