# Supplementary material for: Characterization of super‐enhancer‐associated functional lncRNAs acting as ceRNAs in ESCC
Source: Mol Oncol. 2020 Jun 20;14(9):2203–30. doi: 10.1002/1878-0261.12726 (PMC7463357; doi:10.1002/1878-0261.12726)

A

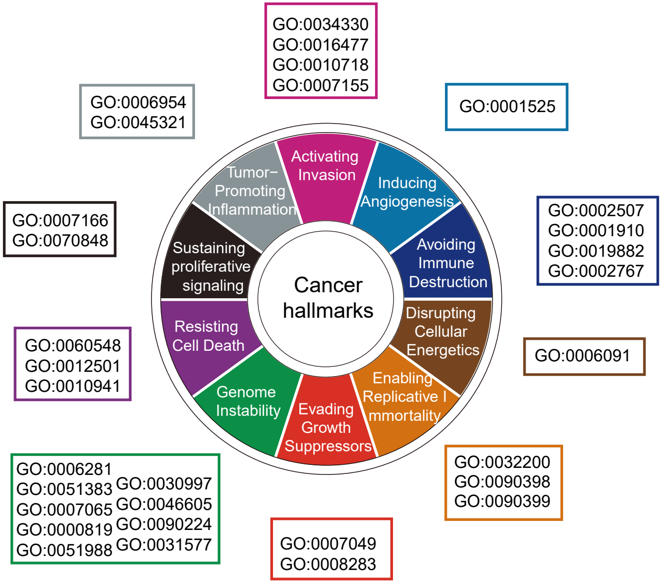

GO term ID	Name of GO terms
GO:0001525	Angiogenesis
GO:0032200	Telomere_organization
GO:0090398	Cellular_senescence
GO:0090399	Replicative_senescence
GO:0034330	Cell_junction_organization
GO:0016477	Cell_migration
GO:0010718	Positive_regulation_of_epithelial_ to_mesenchymal_transition
GO:0007155	Cell_adhesion
GO:0006281	DNA_repair
GO:0051383	Kinetochore_organization
GO:0007065	Sister_chromatid_cohesion
GO:0000819	Sister_chromatid_segregation
GO:0051988	Regulation_of_attachment_of_spindle _microtubules_to_kinetochore
GO:0030997	Regulation_of_centriolecentriole_cohesion
GO:0046605	Regulation_of_centrosome_cycle
GO:0090224	Regulation_of_spindle_organization
GO:0031577	Spindle_checkpoint
GO:0060548	Negative_regulation_of_cell_death
GO:0012501	Programmed_cell_death
GO:0010941	Regulation_of_cell_death
GO:0006091	Generation_of_precursor_metabolites_and_energy
GO:0007166	Cell_surface_receptor_signaling_pathway
GO:0070848	Response_to_growth_factor_stimulus
GO:0006954	Inflammatory_response
GO:0045321	Leukocyte_activation
GO:0002507	Tolerance_induction
GO:0001910	Regulation_of_leukocyte_mediated_cytotoxicity
GO:0019882	Antigen_processing_and_presentation
GO:0002767	Immune_response-inhibiting_cell_surface _receptor_signaling_pathway
GO:0007049	Cell_cycle
GO:0008283	Cell_proliferation

B

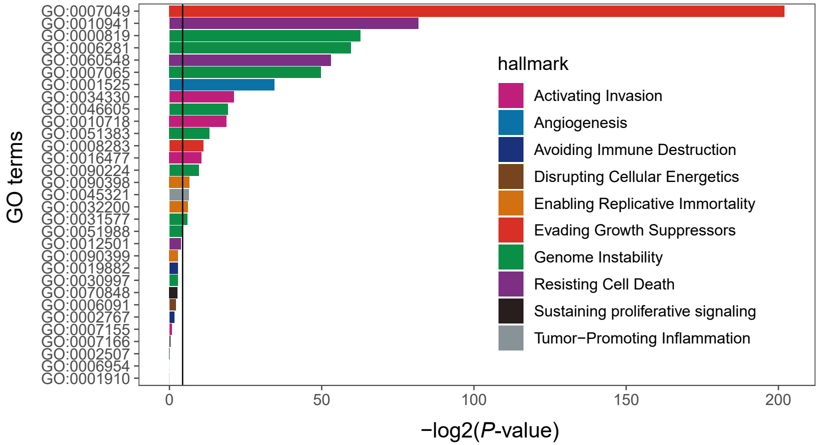

C

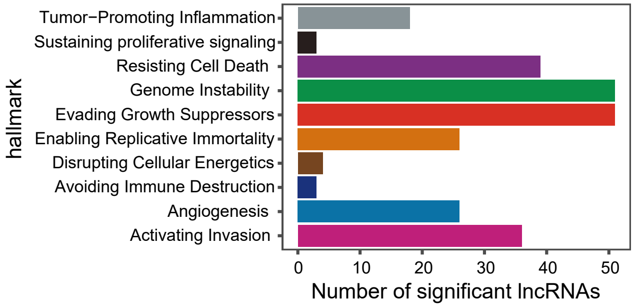

Supplement: Supplementary file 2 — Fig. S2. The ceRNA network controls broad cancer associated hallmarks. (A) The cancer hallmarks corresponding to GO terms. The colors corresponds to different cancer hallmarks. (B) The cancer hallmarks related GO terms enriched by ce‐lncRNA‐related PCGs in the ceRNA network. The colors of bars corresponds to different cancer hallmarks. (C) Number of significantly enriched ce‐lncRNAs for each cancer hallmark. [file MOL2-14-2203-s002.pdf]
