# Supplementary material for: Characterization of super‐enhancer‐associated functional lncRNAs acting as ceRNAs in ESCC
Source: Mol Oncol. 2020 Jun 20;14(9):2203–30. doi: 10.1002/1878-0261.12726 (PMC7463357; doi:10.1002/1878-0261.12726)
Supplement: Supplementary file 6 — Fig. S6. Box plots of log2 fold changes in expression of lncRNA associated ce‐PCGs in KYS510 and TE7 cells treated with either DMSO or THZ1 (50nM) at indicated time points. * P < 0.05, ** P < 0.01, *** P < 0.001. P values were determined using Wilcoxon rank‐sum test. [file MOL2-14-2203-s006.pdf]

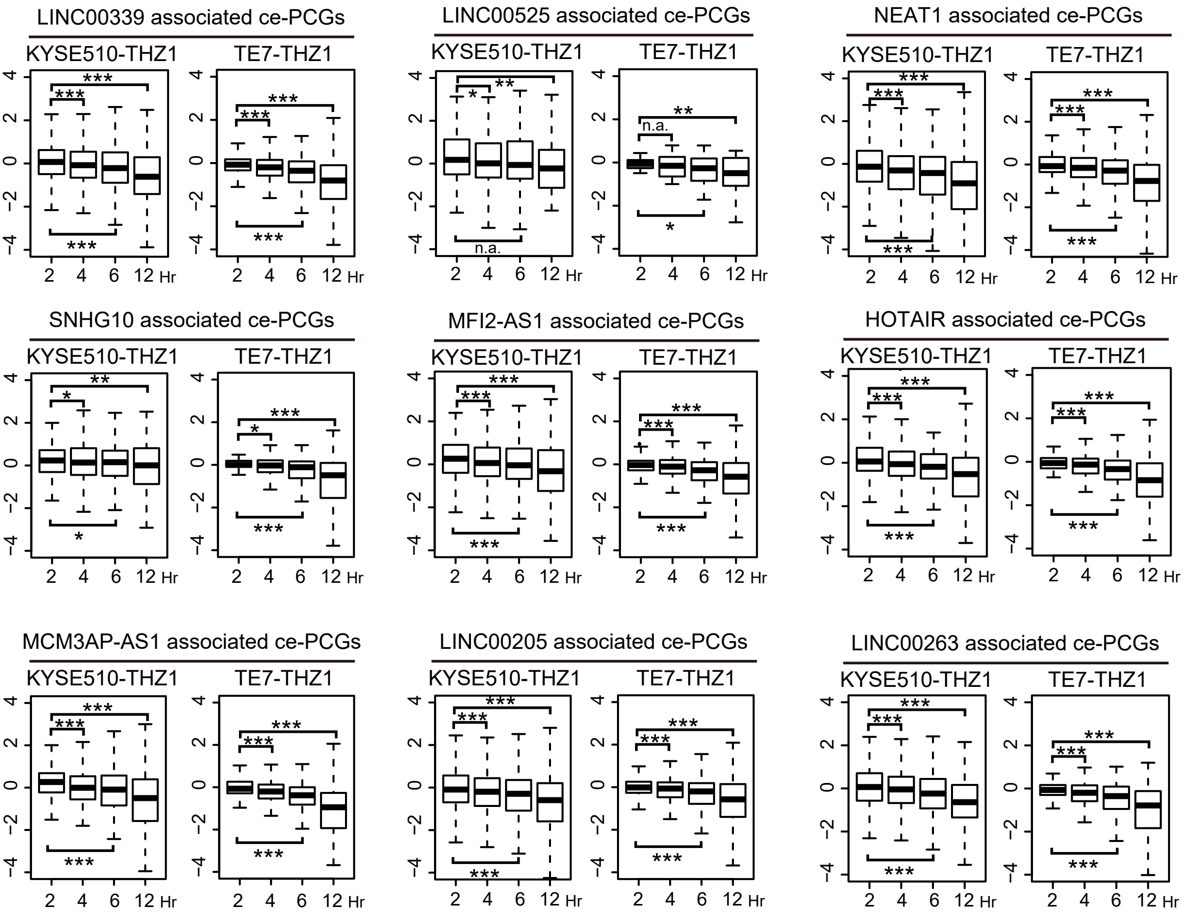
LINC00339 associated ce-PCGs
LINC00525 associated ce-PCGs
NEAT1 associated ce-PCGs
SNHG10 associated ce-PCGs
MFI2-AS1 associated ce-PCGs
HOTAIR associated ce-PCGs
MCM3AP-AS1 associated ce-PCGs
LINC00205 associated ce-PCGs
LINC00263 associated ce-PCGs
KYSE510-THZ1
TE7-THZ1
***
**
*
n.a.
2
4
6
12 Hr
4
2
0
−2
−4
