# Supplementary material for: Characterization of super‐enhancer‐associated functional lncRNAs acting as ceRNAs in ESCC
Source: Mol Oncol. 2020 Jun 20;14(9):2203–30. doi: 10.1002/1878-0261.12726 (PMC7463357; doi:10.1002/1878-0261.12726)
Supplement: Supplementary file 9 — Table S1. Clinical and pathological characteristics of patients in four datasets for genome‐wide gene expression profiles of ESCC. Table S2. An example of calculating local regulatory direction consistency of a potential lncRNA‐PCG ceRNA pair. Table S3. The overlap and similarity of ceRNA pairs and ce‐lncRNAs identified in two ESCC datasets. Table S4. siRNA target sequences. Table S5. Primers used in this study. [file MOL2-14-2203-s009.docx]

**Supplementary Table 1.** Clinical and pathological characteristics of patients in four datasets for genome-wide gene expression profiles of ESCC.

| **Characteristics** | **SRP064894**  **(n=15)** | **GSE53625**  **(n=119)** | **GSE53625**  **(n=60)** | **TCGA ESCC**  **(n=80)** |
| --- | --- | --- | --- | --- |
| **Age (years)** |  |  |  |  |
| ≤59 | 11 (73.3) | 62 (52.1) | 29 (48.3) | 48 (60.0) |
| >59 | 4 (26.7) | 57 (47.9) | 31 (51.7) | 32 (40.0) |
| **Gender** |  |  |  |  |
| Male | 11 (73.3) | 98 (82.4) | 48 (80.0) | 69 (86.3) |
| Female | 4 (26.7) | 21 (17.6) | 12 (20.0) | 11 (13.7) |
| **Vital status** |  |  |  |  |
| Alive | 1(6.7) | 46 (38.7) | 27 (45.0) | 63 (78.8) |
| Dead | 14 (93.3) | 73 (61.3) | 33 (55.0) | 17 (21.2) |
| **Tobacco use** |  |  |  |  |
| Yes | 8 (53.3) | 80 (67.2) | 34 (56.7) | 56 (70.0) |
| **Alcohol use** |  |  |  |  |
| Yes | 4 (26.7) | 74 (62.2) | 32 (53.3) | 58 (72.5) |
| **Disease stage,no** |  |  |  |  |
| **T** |  |  |  |  |
| T1 |  | 8 (6.7) | 48 (80.0) | 7 (8.8) |
| T2 | 2 (13.3) | 20 (16.8) | 7 (11.7) | 23 (28.8) |
| T3 | 13 (86.7) | 62 (52.1) | 4 (6.7) | 46 (57.5) |
| T4 |  | 29 (24.4) | 1 (1.6) | 4 (4.9) |
| **N** |  |  |  |  |
| N0 | 9 (60.0) | 54 (45.4) | 29 (48.3) | 47 (58.8) |
| N1 | 3 (20.0) | 42 (35.3) | 20(33.3) | 24(30.0) |
| N2 | 3 (20.0) | 13(11.0) | 9 (15.0) | 6 (7.5) |
| N3 |  | 10 (8.3) | 2 (3.4) | 2 (3.7) |
| **TNM** |  |  |  |  |
| I | 2 (13.3) | 6 (5.0) | 4 (6.7) |  |
| II | 7 (46.7) | 47 (39.5) | 30 (50.0) |  |
| III | 6 (40.0) | 66 (55.5) | 26 (43.3) |  |

Data are shown as n (%); TNM, tumor node metastasis; ESCC, esophageal squamous cell carcinoma.

**Supplementary Table 2.** An example of calculating local regulatory direction consistency of a potential lncRNA-PCG ceRNA pair.

|  | **Sample 1** | **Sample 2** | **Sample 3** | **Sample 4** | **Sample 5** | **Sample 6** | **...** | **Sample i** | **...** | **Sample 15** |
| --- | --- | --- | --- | --- | --- | --- | --- | --- | --- | --- |
| PCG ($\log_{2}\mathrm{FC}$) | 3.1**↑** | 0.5 | -0.6 | -2.3**↓** | 11.7**↑** | 0.4 | ... | -3.2**↓** | ... | 4.5**↑** |
| LncRNA ($\log_{2}\mathrm{FC}$) | 2.2**↑** | 0.2 | -0.3 | 4.0**↑** | -5.9**↓** | 7.6**↑** | ... | -4.6**↓** | ... | 1.4**↑** |
| $\mathrm{dec}_{i}(l,g)$ | 1 | 0 | 0 | 0 | 0 | 0 | ... | -1 | ... | 1 |
| $DEC-score(l,g)$ | \|1\|+\|0\|+\|0\|+\|0\|+\|0\|+\|0\|+…+\|-1\|+…+\|1\|=3… | | | | | | | | | |

**↑:** $\log_{2}\left( \mathrm{FC}_{l}^{i} \right)$>1 or $\log_{2}\left( \mathrm{FC}_{g}^{i} \right)$>1; **↓:** $\log_{2}\left( \mathrm{FC}_{l}^{i} \right)$<-1 or $\log_{2}\left( \mathrm{FC}_{g}^{i} \right)$<-1

**Supplementary Table 3.** The overlap and similarity of ceRNA pairs and ce-lncRNAs identified in two ESCC datasets.

| **Cutoff** | **CeRNA pairs** | | | | **Ce-lncRNAs** | | | |
| --- | --- | --- | --- | --- | --- | --- | --- | --- |
|  | **SRP064894 number** | **GSE53625 number** | **Overlap**  **number** | **Similarity** | **SRP064894 number** | **GSE53625 number** | **Overlap number** | **Similarity** |
| 3 | 12235 | 18165 | 1929 | 0.067 | 103 | 81 | 72 | 0.64 |
| 4 | 11943 | 15464 | 1695 | 0.065 | 101 | 77 | 68 | 0.61 |
| **5** | **11012** | **13268** | **1365** | **0.059** | **98** | **71** | **61** | **0.56** |
| 6 | 8999 | 11462 | 941 | 0.048 | 96 | 65 | 56 | 0.53 |
| 7 | 6990 | 9891 | 562 | 0.034 | 91 | 64 | 53 | 0.51 |
| 8 | 5521 | 8673 | 379 | 0.027 | 74 | 60 | 38 | 0.39 |
| 9 | 3960 | 7797 | 305 | 0.026 | 60 | 59 | 32 | 0.36 |
| 10 | 3096 | 7033 | 223 | 0.022 | 49 | 58 | 27 | 0.33 |

**Supplementary Table 4.** siRNA target sequences.

| **siRNA** | **Target Sequence（5**′**-3**′**）** |
| --- | --- |
| siNC-1 | UGGUUUACAUGUCGACUAA |
| siNC-2 | UGGUUUACAUGUUGUGUGA |
| siNC-3 | UGGUUUACAUGUUUUCUGA |
| siNC-4 | UGGUUUACAUGUUUUCCUA |
| siLINC00094-1 | AGUCAUUAAUCCUUCGAAA |
| siLINC00094-2 | CCGUUAAUUUAGUGCGCGU |
| siLINC00094-3 | CAUUAGACUGAGCGCAUCA |
| siLINC00094-4 | AUACAGAAGAAACGGACGU |
| siLINC00338-1 | AUAAAUAGAUACACGCCAA |
| siLINC00338-2 | GUGCUAUUUGCCACGGAAA |
| siLINC00338-3 | UGGUACAUUUGAGGGUUAA |
| siLINC00338-4 | CAUCCAGGUUUGUUUGGUU |
| siSNHG10-1 | GCUUGACAAUAUACAGUUA |
| siSNHG10-2 | GCAUAAUUGUUGUUUCAGA |
| siSNHG10-3 | UACUAUUGGUCGUCGGCAA |
| siSNHG10-4 | CAAGAGGGAAGACGACUUU |
| siMFI2-AS1-1 | CCACAAACCUAAACAAUUU |
| siMFI2-AS1-2 | ACCCAAAGAGCAAGUCAUU |
| siMFI2-AS1-3 | CUGGAAGAGGCGUUCAGAA |
| siMFI2-AS1-4 | CCAGAAGUAGUAUGAGAAC |
| siTCF3-1 | GCCUCUCUUCAUCCACAUUTT |
| siTCF3-2 | GCAAUAACUUCUCGUCCAGTT |
| siTCF3-3 | GCGGAACCUGAAUCCCAAATT |
| siKLF5-1 | GCAGACUGCAGUGAAACAATT |
| siKLF5-2 | GGCAAUUCACAAUCCAAAUTT |
| siKLF5-3 | GCAUCCACUACUGCGAUUATT |

**Supplementary Table 5.** Primers used in this study.

| **Primer** | **Sequence（5**′**-3**′**）** |
| --- | --- |
|  | **Primers for qRT-PCR** |
| LINC00094-qF | ACGGGACCAGCACCTTTG |
| LINC00094-qR | GATCCAGTGCCCTGAGGAAG |
| LINC00338-qF | CCCGGGACGATAACAGAGC |
| LINC00338-qR | GCCAATCGTCGGGGGATATT |
| SNHG10-qF | CCTCATCAGGCCCATTGCTT |
| SNHG10-qR | TGTTCATGTTGCTTGTTCCTCT |
| MFI2-AS1-qF | ATTGGCCTCTGTCACCCAAG |
| MFI2-AS1-qR | GGGTGGGTCAGTGAAGACAC |
| TCF3-qF | CCACTCGGAGGAGGAGAAGA |
| TCF3-qR | TTCTCGCTGTTGAGGTGCAG |
| KLF5-qF | GCAGGACGAGCCGGTGTT |
| KLF5-qR | AGGAAGCTGAGGTGTCAGATACT |
| ACTB-qF | CAACTGGGACGACATGGAGAAA |
| ACTB-qR | GATAGCAACGTACATGGCTGGG |
|  | **Primers for ChIP-PCR** |
| LINC00094-E1-qF-1 | GGCAGTGCTCGAGCCAAA |
| LINC00094-E1-qR-1 | CAGGCACTTGCGTGTGTGAA |
| LINC00094-E1-qF-2 | GAGGCCCAAGGATGCGAC |
| LINC00094-E1-qR-2 | GAAGTTGGCCAAAGTCCCCT |
| LINC00094-E2-qF-1 | CTGACAGCACGTTCGTTCCT |
| LINC00094-E2-qR-1 | CCTGGACCACTGAGCTTGAA |
| LINC00094-E2-qF-2 | GGAGTGGGTTGCGATGCTT |
| LINC00094-E2-qR-2 | TCCATCTGTGAAACAGGAGCC |
| LINC00094-E3-qF-1 | ACGCCCCAGCTTTATTCACA |
| LINC00094-E3-qR-1 | GTTGCCGGATTATTGCACCC |
| LINC00094-E3-qF-2 | CCAAGTTCGCTGTTGCTGGA |
| LINC00094-E3-qR-2 | TGGTATCTAGTTCCCGCCCC |

F, forward primer; R, reverse primer.
