# Supplementary material for: Characterization of super‐enhancer‐associated functional lncRNAs acting as ceRNAs in ESCC
Source: Mol Oncol. 2020 Jun 20;14(9):2203–30. doi: 10.1002/1878-0261.12726 (PMC7463357; doi:10.1002/1878-0261.12726)
Supplement: Supplementary file 8 — Fig. S8. The downstream pathway analysis of LINC00094. (A) The pathways significantly enriched by LINC00094‐related PCGs in the ceRNA network. Enrichment significance was performed by the iSubpathwayMiner software package using hypergeometric test. The pathways with FDR corrected P < 0.05 were considered as significant. Number of (*) represents the annotated gene number in the corresponding pathway. (B) LINC00094‐related PCGs that were annotated to the ‘PI3K‐Akt signaling’ pathways. (C) The ‘PI3K‐Akt signaling’ pathways where LINC00094‐related PCGs were annotated. The genes (rectangular nodes) mapped by LINC00094‐related PCGs were shown with red node labels and borders. [file MOL2-14-2203-s008.pdf]

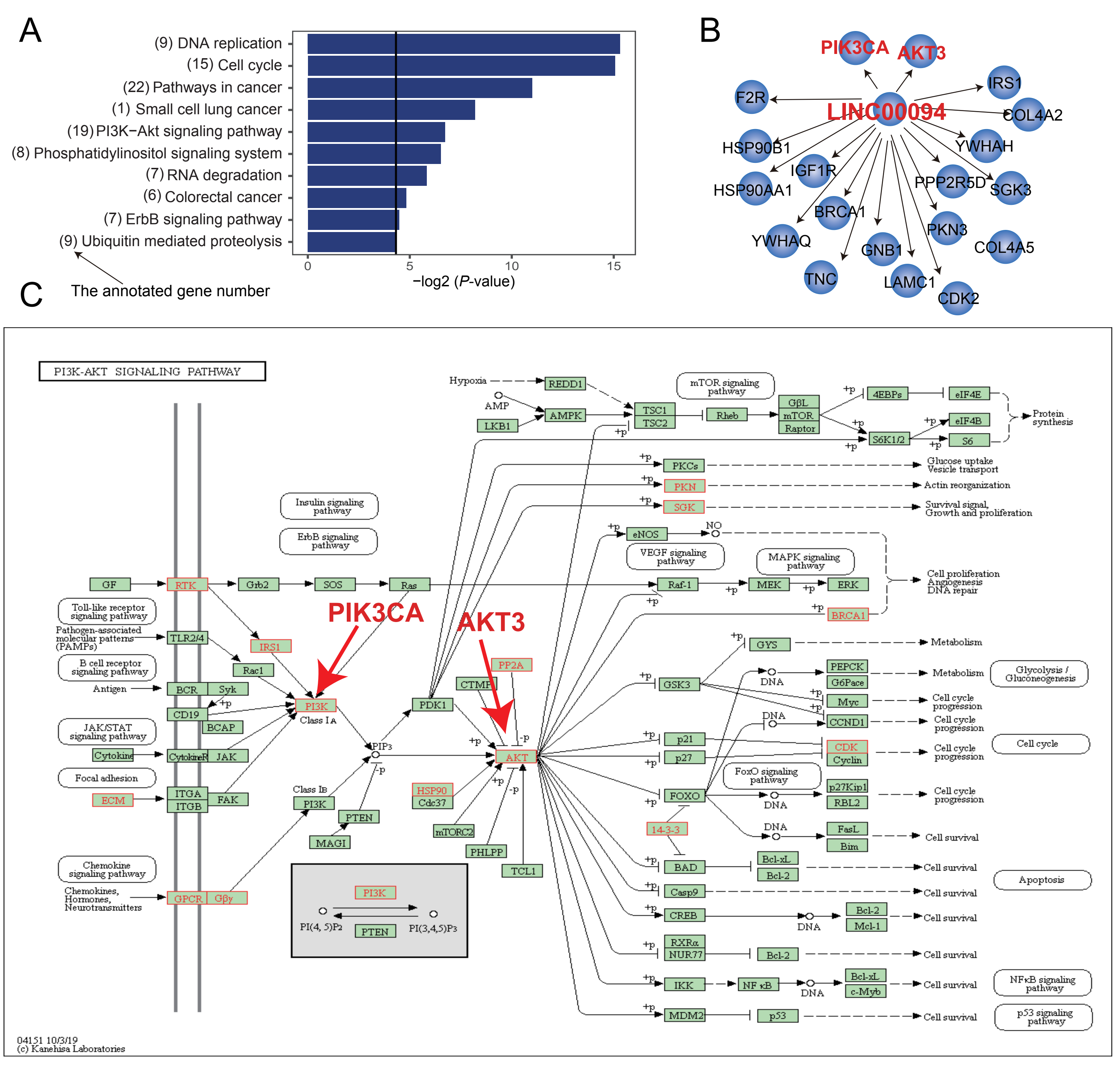

A
(9) DNA replication
(15) Cell cycle
(22) Pathways in cancer
(1) Small cell lung cancer
(19) PI3K−Akt signaling pathway
(8) Phosphatidylinositol signaling system
(7) RNA degradation
(6) Colorectal cancer
(7) ErbB signaling pathway
(9) Ubiquitin mediated proteolysis
0
5
10
15
−log2 (P-value)
The annotated gene number
B
LINC00094
PIK3CA
AKT3
IRS1
COL4A2
F2R
HSP90B1
YWHAH
IGF1R
HSP90AA1
PPP2R5D
SGK3
BRCA1
YWHAQ
PKN3
COL4A5
GNB1
TNC
LAMC1
CDK2
C
PI3K-AKT SIGNALING PATHWAY
PIK3CA
AKT3
Hypoxia
REDD1
AMP
AMPK
LKB1
TSC1
TSC2
mTOR signaling pathway
Rheb
GβL
mTOR
Raptor
4EBPs
eIF4E
eIF4B
S6K1/2
S6
Protein synthesis
PKCs
PKN
SGK
Glucose uptake
Vesicle transport
Actin reorganization
Survival signal,
Growth and proliferation
Insulin signaling pathway
ErbB signaling pathway
eNOS
NO
VEGF signaling pathway
MAPK signaling pathway
GF
RTK
Grb2
SOS
Ras
Raf-1
MEK
ERK
Cell proliferation
Angiogenesis
DNA repair
BRCA1
Toll-like receptor signaling pathway
Pathogen-associated molecular patterns (PAMPs)
TLR2/4
IRS1
Rac1
B cell receptor signaling pathway
Antigen
BCR
Syk
CD19
BCAP
PI3K
Class IA
JAK/STAT signaling pathway
Cytokine
CytokineR
JAK
Focal adhesion
ECM
ITGA
ITGB
FAK
Class IB
PI3K
PTEN
MAGI
PIP3
PDK1
CTMP
PP2A
AKT
HSP90
Cdc37
mTORC2
PHLPP
TCL1
GYS
Metabolism
GSK3
PEPCK
G6Pace
Glycolysis / Gluconeogenesis
Myc
CCND1
Cell cycle progression
p21
p27
CDK
Cyclin
Cell cycle
FoxO signaling pathway
FOXO
p27Kip1
RBL2
14-3-3
FasL
Bim
Cell survival
BAD
Bcl-xL
Bcl-2
Apoptosis
Casp9
CREB
Mcl-1
RXRα
NUR77
IKK
NF κB
c-Myb
NFκB signaling pathway
MDM2
p53
p53 signaling pathway
DNA
Chemokine signaling pathway
Chemokines, Hormones, Neurotransmitters
GPCR
Gβγ
PI(4, 5)P2
PI(3,4,5)P3
04151 10/3/19
(c) Kanehisa Laboratories
